# The Effects of In Utero HIV and Antiretroviral Therapy Exposure on Infant T‐Cell and Monocyte Activation, Function, and Regulation of Immune‐Modulatory Pathways

**DOI:** 10.1155/mi/2928164

**Published:** 2026-03-24

**Authors:** Andrea Prinsloo, Helen C. Steel, Ute Feucht, Theresa M. Rossouw

**Affiliations:** ^1^ Department of Hematology, Faculty of Health Sciences, University of Pretoria, Pretoria, 0001, South Africa, up.ac.za; ^2^ Department of Hematology, National Health Laboratory Service, Tshwane Academic Division, Pretoria, 0001, South Africa, nhls.ac.za; ^3^ Department of Immunology, Faculty of Health Sciences, University of Pretoria, Pretoria, 0001, South Africa, up.ac.za; ^4^ Research Centre for Maternal, Fetal, Newborn and Child Health Care Strategies, University of Pretoria, Pretoria, 0001, South Africa, up.ac.za; ^5^ Department of Pediatrics, Faculty of Health Sciences, University of Pretoria, Pretoria, 0001, South Africa, up.ac.za; ^6^ Maternal and Infant Health Care Strategies Research Unit, South African Medical Research Council, Pretoria, 0001, South Africa, mrc.ac.za

**Keywords:** activation, adaptive, anti-inflammatory, exhaustion, growth, HIV-exposed-uninfected, immunity, infants, innate, pro-inflammatory

## Abstract

Human immunodeficiency virus (HIV) infection is characterized by chronic, systemic immune activation. It is unclear how this affects the immune system of infants born to mothers living with HIV (MLWH). The current study assessed whether maternal HIV status and in utero antiretroviral therapy (ART) exposure impact infant T‐cell and monocyte activation and regulation, as well as monocyte responsiveness to stimulation at birth and early infancy. T‐cell and monocyte activation and expression of immune checkpoint molecules were characterized by means of flow cytometry. Pro‐ and anti‐inflammatory cytokine/chemokine profiles were assessed using a suspension bead array assay. Seventy‐one pregnant MLWH and 77 mothers not living with HIV (MNLWH) were recruited at 22 weeks’ gestation, and mother‐infant pairs were followed until 6 months postpartum. MLWH had higher percentages of CD4+ and CD8+ T‐cells expressing programmed cell death protein‐1 (PD‐1) and a higher percentage of regulatory T‐cells (Tregs). HIV‐exposed‐but‐uninfected (HEU) infants displayed disrupted CD4+ T‐cell maturation with an increased number of CD8+ T‐cells expressing PD‐1 at the time of birth and increased T‐cell exhaustion at 10 weeks of age. Higher levels of monocyte activation were observed in MLWH with increased numbers of classical (CL) monocytes expressing CCR2 and CD80. An increased percentage of CL monocytes expressing CCR2 and CD80 was noted in HEU infants at the time of birth, which persisted at 10 weeks of age. Significantly higher levels of C‐reactive protein (CRP) and non‐significantly higher levels of interleukin (IL)‐6 and tumor necrosis factor‐alpha were observed in MLWH, indicative of hyperactivated innate inflammation. HEU infants had persistently increased levels of IL‐8 and transforming growth factor (TGF)‐β1, and lower levels of IL‐10, IFN‐γ, and CRP. The latter might be secondary to cotrimoxazole use in the HEU infants while the altered cytokine levels might be indicative of altered immune programing. In summary, this cohort study showed that maternal HIV status has a transient effect on basal infant T‐cell and monocyte activation, regulation, and monocyte responsiveness, which dissipates at 6 months of age, while altered cytokine levels persisted.

## 1. Introduction

The introduction of antiretroviral therapy (ART) for all pregnant and breastfeeding women living with the human immunodeficiency virus (HIV) has led to a significant decrease in the number of infants infected with this virus. In 2022, 7.6 million people were living with HIV (PLWH) in South Africa (RSA) and the rate of acquiring new infections was 3.1 per 1000 [1]. A staggering 63% of PLWH in RSA in 2022 were women [1]. In addition, children who are HIV‐exposed‐but‐uninfected (HEU) account for roughly 29% of all births in RSA [2]. Statistics South Africa [3] reported 932,204 live births in 2022. This translates to an estimated 270,339 HEU infants being born in RSA in 2022. Based on an ART coverage rate of 98% in pregnant women, the vast majority of these HEU infants have also been exposed to ART in utero [1]. These children are now well recognized as having persistent health disparities compared with their HIV‐unexposed‐and‐uninfected (HUU) counterparts [4, 5]. Differences reported between these two groups include immune dysfunction [6] and higher levels of inflammation, cognitive [7], and metabolic abnormalities [8], sub‐optimal growth [9], as well as increased morbidity and mortality [5]. The reasons for these disparities remain largely unknown [10].

The human immune response depends on a delicate balance of communication and interaction between the innate and adaptive arms of the immune system. The innate arm is the first line of defense with a non‐specific response against foreign micro‐organisms, while the adaptive arm has a targeted approach and is predominantly responsible for the development of immune memory [11]. Infection with HIV is associated with chronic, systemic immune activation, driven by both the innate and adaptive arms of the immune systems, and which persists at low levels despite virally suppressive ART [12]. The same is true in pregnant women/mothers living with HIV (MLWH) [13]. Furthermore, alterations in the maternal gut microbiome and mucosal dysregulation resulting from HIV infection [14], accompanied by subsequent microbial translocation, contribute to inflammation [5, 13] and changes in the cytokine/chemokine profile of MLWH. It is still unclear if these altered profiles might affect the development of immune cells and the immune responses in HEU infants [12].

For instance, altered cell‐mediated immunity, including impaired T‐cell maturation and hypo‐ as well as hyper‐responsiveness to T‐cell activation, has been reported in HEU infants but remain incompletely understood [15].

Furthermore, evidence suggests that HEU infants possess reduced diversity in the T cell receptor beta chain, indicating a potential impairment in the adaptive immune system [16]. Additionally, the mechanisms underlying increased susceptibilities to infections, particularly from encapsulated bacteria and certain viruses, remain undefined, suggesting deficiencies in both humoral and possibly cell‐mediated immune responses [17]. Although a decreased transplacental transfer of maternal antibodies has been observed, a comprehensive functional analysis of antibody and cellular immune responses in HEU infants, especially across varying severities of maternal disease, is still required [18].

Moreover, while some perturbations in T‐cell subsets have been correlated with maternal HIV viral load (VL), the precise influence of these factors on HEU infant health outcomes remains unclear [19]. This complexity is further compounded by conflicting data on whether prenatal exposure to HIV‐associated pro‐inflammatory signals contributes to the immunological profiles observed at birth and how this might affect the infants’ susceptibility to infections later in life [20].

In summary, while some progress has been made in uncovering the immune system’s nuances in HEU infants, crucial unknowns about the mechanisms leading to altered immunity and increased vulnerability to infections and developmental issues remain [21]. Further research is necessary to elucidate these mechanisms and improve interventions for this vulnerable population. The current study therefore explored the effects of in utero HIV and ART exposure on infant T‐cell and monocyte activation, function, and regulation of immune‐modulatory pathways.

## 2. Materials and Methods

### 2.1. Participants

South‐West Tshwane in RSA records about 10,000 births annually from 14 antenatal clinics and three delivery sites, including Kalafong Provincial Tertiary Hospital, which offers specialized antenatal and obstetric care. Approximately 21% of pregnant women in this area are living with HIV and the region has a vertical transmission rate below 2% [22]. HIV counseling and testing are routinely offered to all pregnant and breastfeeding women with unknown HIV status or those who tested negative more than 3 months previously.

All women confirmed to be living with HIV are eligible for immediate initiation of lifelong ART and, at the time of recruitment for the current study, MLWH were started on a fixed‐dose combination of tenofovir disoproxil fumarate, emtricitabine, and efavirenz.

The present study is part of the larger Siyakhula study, a prospective, longitudinal, descriptive cohort study initiated in 2018 by clinicians and researchers at the Maternal and Infant Health Care Strategies Unit of the University of Pretoria, RSA. The Siyakhula study recruited 315 dyads (152 MLWH and their HEU infants, and 163 pregnant women/mothers not living with HIV [MNLWH] and their HUU infants) from antenatal clinics in Southwest Tshwane, RSA, before 22 weeks’ gestation. Follow‐up visits were at 28‐ and 36‐weeks’ gestation, birth, 6, 10, 14, and 24 weeks, and 1 and 2 years of age.

The current study included 76 consecutive pregnant women (42 MLWH and 34 MNLWH) recruited as part of the Siyakhula study described above. Flow cytometry and multi‐analyte cytokine/chemokine analyses were conducted on these pairs of participants at 28 weeks of pregnancy, birth, 10 weeks, and 6 months of age. Anthropometric measurements taken at 22 weeks’ gestation (baseline) and at birth, 10 weeks, 6, and 12 months postpartum from a larger subset of mother/infant pairs from the Siyakhula study cohort including 76 MLWH and 79 MNLWH were analyzed in combination with the flow cytometry and multi‐analyte cytokine assay data. The Siyakhula study (294/2017) and the current study (863/2019) were approved by the Research Ethics Committee of the Faculty of Health Sciences at the University of Pretoria, RSA and conducted in accordance with the principles outlined in the Declaration of Helsinki. Written informed consent was obtained from all participants.

The number of study participants fluctuated between the various time points. These variations were primarily attributed to: (i) some infants not being delivered at Kalafong Tertiary Academic Hospital due to the mothers entering labor before reaching the hospital; (ii) some participants missing certain study visits; and iii) insufficient blood drawn at a particular visit, particularly in the case of infants. Furthermore, the COVID‐19 pandemic restricted the number of participants able to access the study site for the 10‐week and 6‐month follow‐up visits, resulting in a smaller sample size than initially projected for these time points. Some participants who missed the 10‐week visit were successfully tracked and returned for subsequent follow‐up visits. A formal sample size calculation was not performed, as the number of experimental procedures was contingent upon the funding obtained.

Exclusion criteria for recruitment into the study included the inability to obtain informed consent, maternal hypertension, diabetes, tuberculosis, or other serious pre‐existing medical conditions, multiple pregnancies, and fetal chromosomal or structural abnormalities.

### 2.2. Clinical Information

In the Siyakhula study, pregnancy, birth, and postpartum clinical data were collected through repeated follow‐up visits. Fetal growth parameters, including bi‐parietal diameter, head circumference (HC), abdominal circumference, femur length, and fetal weight estimation according to the methods described by Hadlock, [23] were measured. Key maternal variables recorded during pregnancy and at delivery included duration of gestation, anthropometric data, and coinfections. For MLWH, CD4+ T‐cell counts, and VLs were determined as per the national guidelines for RSA [24].

Medical information recorded at birth included infant weight, length, HC, and placental weight. Postpartum variables included infant growth (weight, height, HC, mid‐upper arm circumference [MUAC]), infectious episodes, and HIV test results. Sex‐ and age‐adjusted Z‐scores were calculated using INTERGROWTH‐21st [25], and World Health Organization (WHO) Anthro online applications [26], adjusted for gestational age (GA). Data were recorded on the REDCap (Research Electronic Data Capture) web application (Vanderbilt University, Nashville, TN, USA) managed by staff based at the South African Medical Research Council unit at Kalafong Provincial Tertiary Hospital, RSA.

### 2.3. Multiparameter Flow Cytometry

The DURAClone IM T‐cell subsets kit comprising the C–C motif chemokine receptor (CCR)7, CD3, CD4, CD8, CD27, CD28, CD45, CD45 receptor antagonist (RA), CD57, and programmed cell death protein‐1 (PD‐1) (Beckman Coulter, Brea, CA, USA) [27], and the DURAClone IM Treg kit consisting of CD3, CD4, CD25, CD39, CD45RA, forkhead box P3 (FoxP3), Helios, and CD45 (Beckman Coulter) [28] were used to characterize and quantify CD4+ and CD8+ T‐cells, as well as Treg subsets from fresh whole blood collected in tubes containing ethylenediaminetetraacetic acid as anticoagulant (Becton, Dickinson and Company, NJ, USA). Results were acquired on a CytoFLEX flow cytometer (Beckman Coulter). The monocyte panel used included CCR2, CD14, CD16, CD80, CD86, programmed cell death ligand (PD‐L)1, PD‐L2, intracellular toll‐like receptor (TLR)3, and TLR4.

All flow cytometry data analysis was performed using the Kaluza and Cytobank software (Beckman Coulter) and captured in Microsoft Excel spreadsheets. Examples of multiparameter flow cytometry data analysis are included in Figure S1 of the supplementary data.

### 2.4. Cytokine/Chemokine Assays

The circulating concentrations of the selected cytokines/chemokines were determined for pregnant women at 28 weeks’ gestation, and infants at 10 weeks, and 6 months of age to characterize the inflammatory profiles. Cytokines/chemokines (granulocyte‐macrophage‐colony stimulating factor [GM‐CSF], interferon‐gamma [IFN‐γ], IL‐2, IL‐4, IL‐6, IL‐8, IL‐10, and tumor necrosis factor‐alpha [TNF‐α]) were measured in stored plasma samples using a customized multi‐analyte MILLIPLEX MAP Human Cytokine/Chemokine Magnetic Bead Panel (Merck, Darmstadt, DE) as instructed by the manufacturer. Analysis was performed on a Bio‐Plex Luminex 200 Suspension Array System (Bio‐Rad Laboratories, Inc., Hercules, CA, USA). Bio‐Plex Manager Software 6.0 was used for bead acquisition and analysis of the median fluorescence intensity. The results are presented in pg/mL.

### 2.5. Enzyme‐Linked Immunosorbent Assays

Transforming growth factor‐beta 1 (TGF‐β1) and soluble CD14 (sCD14) were measured using individual enzyme‐linked immunosorbent assay (ELISA) kits supplied by Elabscience (Elabscience, Houston, TX, USA). Neopterin was measured using the Human Neopterin (NPT) ELISA Kit (Abbexa, Houston, TX, USA). The protocols for all ELISAs were followed as described by the manufacturer. The optical density of the ELISA assays was measured using a spectrophotometer (PowerWaveX; BioTek Instruments Inc., Winooski, VT, USA) set at a wavelength of 450 nm. Results are presented as ng/mL.

### 2.6. C‐Reactive Protein (CRP) Analysis

CardioPhase hsCRP (Siemens, Munich, DE) was used to quantitatively measure the acute phase reactant, CRP, in plasma samples using the Attelica 630N nephelometer (Siemens). Results are presented as mg/L.

### 2.7. Statistical Analysis

Clinical and immunological data were exported to Stata18 (StataCorp, College Station, TX, USA) for analysis. Data were initially inspected with histograms to identify outliers and improbable results. The Shapiro–Wilk test determined data distribution. Normally distributed continuous data were summarized as means and standard deviations (SD), with independent groups compared using the Student’s *t*‐test. Non‐normally distributed variables were described with medians and interquartile ranges (IQR) and compared using the Kruskall‐Wallis and post‐hoc Dunn tests.

Categorical variables were expressed as proportions, frequencies, and percentages, and compared with Pearson’s chi‐square or Fisher’s exact tests, as appropriate. Correlations were assessed by means of the non‐parametric Spearman correlation test with Bonferroni correction for multiple comparisons. A *p*‐value of 0.05 was taken as significant and a rho >0.75 as a strong correlation. Within‐group changes over time were assessed using the paired *t*‐test or Wilcoxon signed‐rank test. A *p*‐value of <0.05 was considered significant. Linear regression was used to test associations with continuous outcome variables, while logistic regression was used to analyze associations with dichotomous outcome variables. Univariate, stepwise, backward, and multivariable regression analyses were conducted. Variables were transformed before model entry, with a 10% *p*‐value threshold for inclusion in the multivariable model.

## 3. Results

### 3.1. Maternal and Infant Anthropometric Findings

A summary of the baseline maternal and infant demographic and anthropometric characteristics is provided in Table 1. Additional infant anthropometric data between 10 weeks and up to 12 months of age are included in Table S1 of the supplementary data. The MLWH were significantly older (*p* = 0.0001) and taller (*p* = 0.0071) but had a lower body mass index (BMI) (*p* = 0.0180) and a smaller MUAC (*p* = 0.0143).

**Table 1:** Baseline maternal and infant demographic and anthropometric characteristics.

Variable	MLWH (*n* = 76)	MNLWH (*n* = 79)	*p*‐Value
Age (Years)	37 (34–39)	33 (26–37)	**0.0001**
Weight (kg)	68.3 (±12.0) ^∗^	65.4 (±12.0) ^∗^	0.0850
Height (m)	1.61 (1.56–1.66)	1.59 (1.54–1.63)	**0.0071**
BMI (kg/m^2^)	26.3 (±4.6) ^∗^	25.9 (±5.1) ^∗^	0.2786
MUAC (cm)	27.6 (±4.2) ^∗^	27.1 (±3.8) ^∗^	0.3069

	**HEU (*n* = 70)**	**HUU (*n* = 71)**	***p*-Value**

Gestational age (Weeks)	36 (±5.7) ^∗^	38 (±3.34) ^∗^	0.2089
Gender (M:F)	35:36	37:34	0.737
Birth weight (g)	2800 (±500) ^∗^	3100 (±500) ^∗^	**0.0022**
Birth weight Z‐score	0.15 (−0.86–0.99)	0.34 (−0.54–1.13)	0.9798
Birth length (cm)	49.5 (±2.9) ^∗^	50.0 (±2.3) ^∗^	0.4907
Birth length Z‐score	1.01 (±1.87) ^∗^	1.03 (±1.50) ^∗^	0.9816
Birth HC (cm)	33.9 (±1.7) ^∗^	34.6 (±1.5) ^∗^	**0.0047**
Birth HC Z‐score	1.25 (0.01–2.01)	1.21 (0.49–1.93)	0.2767

*Note:* Results are presented as median and interquartile range, except where an asterisk ( ^∗^) indicates that results are presented as mean and standard deviation. *p*‐values denoted in bold indicate significance (*p*  < 0.05).

Abbreviations: BMI, body mass index; cm, centimeters; F, female; g, grams; HC, head circumference; HEU, HIV‐exposed‐uninfected; HUU, HIV‐unexposed‐uninfected; kg, kilograms; M, male; m, meters; m^2^, square meters; MLWH, mothers living with HIV; MNLWH, mothers not living with HIV; MUAC, mid‐upper arm circumference.

The HEU infants weighed significantly less at birth (*p* = 0.0022), 10 weeks (*p* = 0.0290) and 6 months of age (*p* = 0.0051) compared to HUU infants. The HC of the HEU infants was significantly smaller at birth (*p* = 0.0047) and 6 months (*p* = 0.0161) while the MUAC was smaller at 6 months of age (*p* = 0.0116). None of these anthropometric differences were evident at 12 months of age.

No difference in length was observed between the HEU and HUU infant groups at any time point. Z‐scores were similar at birth, but the BMI‐for‐age Z‐score (BAZ) was significantly smaller in HEU infants at 10 weeks (*p* = 0.0366) and 6 months (*p* = 0.0339), while the weight‐for‐length Z‐score (WLZ) (*p* = 0.0156) and weight‐for‐age Z‐score (WAZ) (*p* = 0.0369) were also smaller in HEU infants by 6 months. No difference in the length‐for‐age Z‐score (LAZ) was observed at any time point. However, at 12 months, significantly lower WLZ (*p* = 0.0227), BAZ (*p* = 0.0262) and HC Z‐score (HCZ) (*p* = 0.0420) were observed in the HEU infants compared to the HUU infant group.

### 3.2. Maternal Antenatal CD4 Count and HIV VLs

MLWH had a median CD4+ T‐cell count of 462 (IQR 273–736) cells/mm^3^ at recruitment which did not change significantly by 28 weeks’ gestation (450 [IQR 283–736] cells/mm^3^; *p* = 0.5078) or at the time of birth (443.5 [IQR 234.5–686] cells/mm^3^; *p* = 0.6953).

VL results were available for 10 MLWH at 22 weeks’ gestation, 12 MLWH at 28 weeks’, and 15 MLWH at birth. In total, 23/76 (30.3%) of MLWH had a VL result for at least one time point. The median VL at 22 weeks’ gestation was 406 (IQR 123 – 2293) copies/mL. A detectable VL (defined as VL >1000 copies/mL) was recorded in four MLWH at 22 weeks’ gestation, eight MLWH at 28 weeks’ gestation and four MLWH at the time of birth. Using a stricter definition of VL >50 copies/mL, increased the number of MLWH with a detectable VL to 10 MLWH at 22 weeks, 15 MLWH at 28 weeks’ gestation, and 12 MLWH at the time of birth. Of these MLWH, five had a persistently elevated VL >1000 copies/mL and 12 had a VL >50 copies/mL.

Maternal CD4 count and VL were moderately and negatively correlated at 28 weeks gestation (rho = −0.58, *p* = 0.0385). MLWH with CD4+ T‐cell counts of <200 cells/mm^3^ had significantly lower weight at 22 weeks’ gestation (*p* = 0.0030) while MLWH with a VL >1000 copies/mL had a lower MUAC at 28 weeks’ gestation (*p* = 0.0041). CD4 count at 28 weeks was positively correlated with maternal weight (rho = 0.44, *p* = 0.0008), MUAC (rho = 0.54, *p* = 0.0333) and BMI (rho = 0.36, *p* = 0.0046) at that time point.

Maternal CD4 count was not correlated with infant anthropometry at any time point but maternal VL (investigated as a VL at any antenatal time point) was strongly and negatively correlated with infant weight at 12 months (rho = −0.89, *p* = 0.0259). Since the numbers were limited (*n* = 6) this result should be interpreted with caution.

### 3.3. Maternal and Infant T‐Cell Profiles

#### 3.3.1. CD4+ and CD8+ T‐Cell Maturation

Figure 1A illustrates the stages of T‐cell maturation from proliferation to senescence with the observed differences between MLWH and MNLWH highlighted. At 28 weeks’ gestation, the total percentage of CD4+ T‐cells was significantly lower in MLWH (*p* = 0.0001) with more effector memory (EM) (*p* = 0.0437), fewer EM1 (*p* = 0.0008), more EM3 (*p* = 0.0117) and terminally differentiated EM cells re‐expressing CD45RA (TEMRA) subtype pE1 (*p* = 0.0330) CD4+ T‐cells found when compared to MNLWH (blue gray arrows). This observation persisted at the time of birth with a decreased proportion of central memory (CM) CD4+ T‐cells also observed (ice blue arrows).

Figure 1Stages of CD4+ and CD8+ T‐cell maturation and function indicating differences between mothers living with and without HIV (A) as well as between HIV‐exposed and‐unexposed infants (B). (Adapted from Koch et al. [29] reproduced under CC BY 2.0 US license) A) Significant differences in CD4+ T‐cell populations and CD8+ T‐cell populations between MLWH and MNLWH at 28 weeks (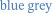 CD4+, 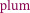 CD8+) and at the time of birth (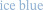 CD4+, 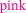 CD8+). B) Statistically significant differences in CD4+ and CD8+ T‐cell populations between HEU and HUU infants at birth (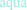 CD4+, 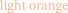 CD8+), 10 weeks of age (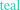 CD4+, 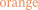 CD8+) and 6 months of age (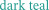 CD4+). Abbreviations: CCR, C–C motif chemokine receptor; CD, cluster of differentiation; CM, central memory; EM, effector memory; N, naïve; T, T‐cell; TEMRA, terminally differentiated effector memory cells re‐expressing CD45RA: E (CD27−CD28−), pE1 (CD27+CD28+), pE2 (CD27+CD28−).

**Figure:**
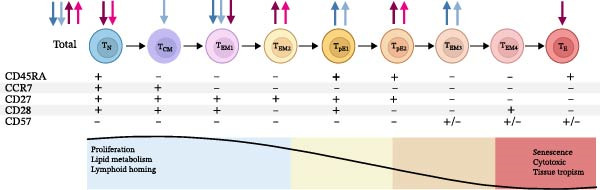
(A)

**Figure:**
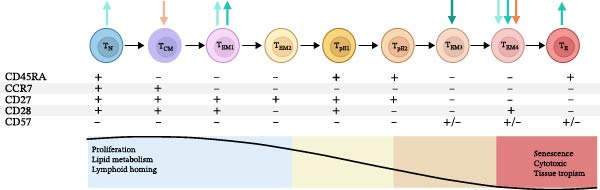
(B)

MLWH had significantly increased numbers of CD8+ cytotoxic T‐cells (*p* = 0.0001) at 28 weeks’ gestation. The proportion of CD8+ naïve (N) T‐cells was significantly lower (*p* = 0.0003) and CD8+ EM T‐cells higher (*p* = 0.0157) with a lower proportion EM1 CD8+ T‐cells (*p* = 0.0066), increased EM2 CD8+ T‐cells (*p* = 0.0045), including a reduced number of CD8+ TEMRA subtype E T‐cells (*p* = 0.0058). In addition, an increased number of TEMRA subtype pE2 CD8+ T‐cells (*p* = 0.0495) was noted (plum arrows). The higher number of CD8+ cytotoxic T‐cells, lower proportion of CD8+ N, and increased proportion of CD8+ EM T‐cells persisted at the time of birth (pink arrows).

No significant differences were noted between the infant groups regarding the total number of CD4+ T‐cells at birth. However, the proportion of CD4+ N (*p* = 0.0476) and CD4+ EM1 T‐cells (*p* = 0.0376) was higher, while the proportion of CD4+ EM4 T‐cells was lower (*p* = 0.0262) in the HEU infants at birth compared to the HUU infants (Figure 1B, aqua arrows). At 10 weeks of age, the increased proportion of CD4+ EM1 T‐cells and decreased proportion of CD4+ EM4 T‐cells persisted, while a higher proportion of TEMRA subtype E CD4+ T‐cells (*p* = 0.0478) was also noted in HEU infants (Figure 1B, teal arrows). At 6 months of age, the proportion of CD4+ EM T‐cells was significantly lower (*p* = 0.0437) in HEU infants; specifically, the proportion of EM3 CD4+ T‐cells (*p* = 0.0280) (Figure 1B, dark teal arrows).

Although the total percentage of CD8+ T‐cells neither differed significantly between the infant groups nor changed significantly over time, significant differences were observed in some CD8+ T‐cell subpopulations. Specifically, HEU infants had decreased proportions of CD8+ CM T‐cells (*p* = 0.0171) at birth (Figure 1B, light orange) and CD8+ EM4 T‐cells (*p* = 0.0243) at 10 weeks of age. Additional maternal and infant CD4+ and CD8+ T‐cell maturation data are included in Tables S2–S5 of the supplementary data.

#### 3.3.2. CD4+ and CD8+ T‐Cell Activation and Exhaustion

In the current study, MLWH presented a highly activated immune environment with a much higher proportion of total CD4+ T‐cells (*p* = 0.0005), including the maturation subsets N, CM, EM, EM1, EM3, and EM4, expressing PD‐1 at 28 weeks’ gestation (Figure 2A, blue gray). This persisted at the time of birth, with increased expression of PD‐1 noted on CD4+ T‐cell subsets N, CM, EM2 and TEMRA subtypes pE1 and E of MLWH (Figure 2A, ice blue). The MLWH also exhibited higher levels of T‐cell exhaustion: at 28 weeks’ gestation, MLWH had increased CD57 expression on the total percentage of CD4+ T‐cells (*p* = 0.0271), CM, EM, EM4, and TEMRA subtype pE1 T‐cells (Figure 2A, blue gray). This pattern persisted at the time of birth, with increased CD57 expression on the total percentage of CD4+ T‐cells (*p* = 0.0124), including N and EM, specifically EM2 T‐cells (Figure 2A, ice blue).

Figure 2Differences in CD4+ and CD8+ T‐cell activation and exhaustion stages in mothers living with and without HIV (A) as well as between HIV‐exposed and‐unexposed infants (B). (Adapted from Koch et al. [29] reproduced under CC BY 2.0 US license) A) Significant differences in CD4+ T‐cell populations and CD8+ T‐cell populations between MLWH and MNLWH at 28 weeks (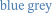 CD4+, 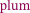 CD8+) and at the time of birth (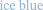 CD4+, 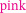 CD8+). B) Significant differences in CD4+ T‐cell populations and CD8+ T‐cell populations between HEU and HUU infants at birth (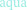 CD4+, 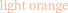 CD8+), 10 weeks of age (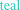 CD4+, 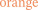 CD8+) and 6 months of age (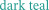 CD4+, 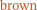). Abbreviations: CCR, C–C motif chemokine receptor; CD, cluster of differentiation; CM, central memory; EM, effector memory; N, Naïve; T, T‐cell; TEMRA, terminally differentiated effector memory cells re‐expressing CD45RA: E (CD27−CD28−), pE1 (CD27+CD28+), pE2 (CD27+CD28−).

**Figure:**
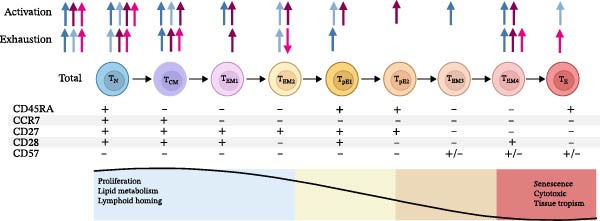
(A)

**Figure:**
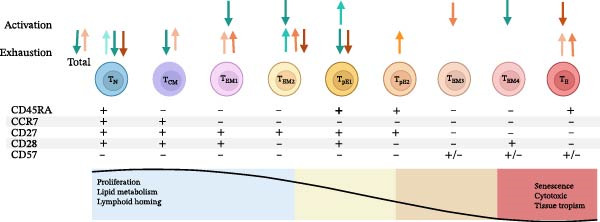
(B)

In contrast, no significant differences in PD‐1 expression on the total percentage of CD4+ T‐cells were observed between HEU and HUU infants at birth. However, increased PD‐1 expression was observed on the TEMRA subtype pE1 CD4+ T‐cells (*p* = 0.0457) at 10 weeks (Figure 2B, teal), with decreased PD‐1 expression on CD4+ EM T‐cells including: EM1, EM2, and EM4, at 6 weeks (Figure 2B dark teal).

The pattern of CD57 expression also changed throughout the study period.

Following increased expression on CD4+ T‐cells, including CD4+ N T‐cells (*p* = 0.0397) at birth (Figure 2B, aqua), and an increased expression on CD4+ EM2 T‐cells (*p* = 0.0236) at 10 weeks (Figure 2B, teal), expression was lower on the total proportion of CD4+ T‐cells (*p* = 0.0026), including N, CM, and TEMRA subtype pE1, at 6 months of age (Figure 2B, dark teal).

MLWH also showed signs of CD8+ T‐cell activation with increased expression of PD‐1 on the total population of CD8+ T‐cells (*p* = 0.0001) as well as on different stages of maturation including N, CM, EM1, EM2, EM4, and TEMRA subtypes pE1 and pE2 at 28 weeks’ gestation (Figure 2A, plum). Increased expression on the total number of CD8+ T‐cells (*p* = 0.0117), including on N CD8+ T‐cells (*p* = 0.0001), persisted in MLWH at the time of birth (Figure 2B, pink). MLWH also had higher levels of CD8+ T‐cell exhaustion with higher expression of CD57 on the N, CM, EM1, and EM4 CD8+ T‐cell subsets at 28 weeks’ gestation (Figure 2A, plum) and on total CD8+ T‐cells (*p* = 0.0367), N, CM, EM4, and TEMRA subtype E, but reduced expression on EM2 CD8+ T‐cells at the time of birth (Figure 2A, pink).

In HEU infants, while no significant differences in PD‐1 expression on the total number of CD8+ T‐cells or any of the CD8+ T‐cell maturation stages were observed at birth, a decreased proportion of CD8+ EM3 T‐cells expressing PD‐1 (*p* = 0.0313) was noted at 10 weeks (Figure 2B, orange) and a reduced number of CD8+ TEMRA subtype E T‐cells expressing PD‐1 (*p* = 0.0066) was observed at 6 months of age (Figure 2B, brown). The HEU also initially had evidence of CD8+ T‐cell exhaustion with higher CD57 expression on the total number of CD8+ T‐cells (*p* = 0.0046), including CM, EM, EM1 and TEMRA subtype E, at birth (Figure 2B, light orange), higher CD57 expression on EM1 (*p* = 0.0184) and TEMRA subtype E CD8+ T‐cells (*p* = 0.0040) at 10 weeks of age (Figure 2B, orange). However, by 6 months, a lower proportion of N (*p* = 0.0402) and EM2 CD8+ T‐cells (*p* = 0.0495) were found to express CD57 in HEU infants (Figure 2B, brown). Additional maternal and infant CD4+ and CD8+ T‐cell activation and exhaustion data are included in Tables S6–S13 of the supplementary data.

#### 3.3.3. CD4+ T‐Cell Regulation

MLWH had a higher proportion of Treg cells at 28 weeks’ gestation (*p* = 0.0080) but no differences were noted in the expression of CD39, CD45RA, and Helios on these cells compared to the MNLWH. In contrast, there was no difference in the proportion of Tregs between the infant groups at any time point and, while no difference was observed in the proportions of CD39+ or Helios+ Tregs, the proportion of CD45RA+ Tregs was significantly lower in HEU infants (*p* = 0.0171) compared to their HUU counterparts at birth.

In addition, CD39 expression on Tregs of HEU infants decreased significantly between birth and 10 weeks of age (*p* = 0.0312). Additional maternal and infant CD4+ T‐cell regulation data are included in Tables S14, S15 of the supplementary data.

### 3.4. Monocyte Profiles

#### 3.4.1. Phenotypic Profiles

No differences were observed in the total percentage of classical (CL) or non‐classical (NC) monocytes between MLWH and MNLWH at 28 weeks’ gestation. However, MLWH had an increased total percentage of intermediate (IM) monocytes (*p* = 0.0105), which persisted at the time of birth. To assess the balance between monocyte subgroups, the monocyte ratio was calculated as follows: CL/(IM + NC). At 28 week’s gestation, there was no difference in the ratios between MLWH and MNLWH, but this had changed at the time of birth with MLWH having significantly lower ratios than MNLWH (*p* = 0.0482) (Table 2). While no differences were observed in the total percentage of CL, IM, and NC monocytes between HEU and HUU infants at birth or 6 months of age, HEU infants had a lower percentage of total NC monocytes (*p* = 0.0406) at 10 weeks of age. No significant differences were observed in the monocyte ratios of the infants at birth, 10 weeks, or 6 months of age (Table 2).

**Table 2:** Summary of phenotypic changes observed in monocytes in mothers living with and without HIV as well as between HIV‐exposed and‐unexposed infants at all time points tested.

Time points	Monocyte ratios median (IQR) (%)			Differences observed		
	MLWH	MNLWH	*p*‐Value			
M28W	4.18 (3.03–4.95)	3.55 (2.64–4.66)	0.3349	↑CCR2	↑Total ↑CCR2 ↑CD86 ↑TLR3 PD‐L1↓	↑CD80
M0W	2.82 (2.01–4.04)	4.68 (2.20–7.84)	0.0482	↓Total ↑CD80 ↑PD‐L1/2	↑Total ↑CCR2 ↑CD86 ↑TLR3	No significant change
	HEU	HUU	*p*‐Value			
B0W	4.89 (2.60–10.22)	5.47 (2.87–8.48)	0.7067	No significant change	↑TLR3	No significant change
B10W	3.65 (2.60–6.95)	3.38 (1.74–8.76)	0.5346	↑CCR2 ↑CD80 ↓TLR4	↓CCR2	↓Total ↓CD86
B6M	3.07 (2.09 –5.21)	2.89 (1.71–4.08)	0.4183	No significant change	No significant change	No significant change
				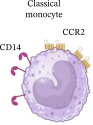	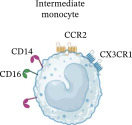	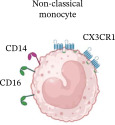
Monocyte Characteristics	Frequency			±90%	2%–8%	2%–11%
	Longevity			1 day	4 days	7 days
	Chemokine signaling			CCR2/CCL2	CCR2/CCL2 CX3CR1/CX3CL1	CX3CR1/CX3CL1
	Roles			Infection control and regulation of inflammation	Pro‐inflammatory	Tissue repair and removal of damaged/dead cells

*Note:* Inserted image adapted from Fu and Drummond [30]. Created in BioRender.

Abbreviations: B0W, babies at birth; B10W, babies at 10 weeks of age; B6M, babies at six months of age; CCL, C‐C motif chemokine ligand; CCR, C‐C motif chemokine receptor; CD, cluster of differentiation; CX3CL, C‐X3‐C motif chemokine ligand; CX3CR, C‐X3‐C motif chemokine receptor; M0W, mothers at the time of birth; M28W, mothers at 28 weeks’ gestation; PD‐L, programmed cell death ligand; TLR, toll‐like receptor.

#### 3.4.2. Activation Status

Table 2 summarizes the phenotypic changes observed in the maternal and infant monocyte subsets. At 28 weeks’ gestation, a higher percentage of total IM monocytes (*p* = 0.0105) expressed CCR2, CD86, and TLR3 in MLWH. However, the number of IM monocytes expressing PD‐L1 was significantly lower in MLWH (*p* = 0.0476). A higher percentage of NC monocytes expressed CD80 (*p* = 0.0103). A lower total number of CL monocytes and a higher number of total IM monocytes were observed in MLWH (*p* = 0.0477) at the time of birth resulting in a low monocyte ratio. The number of CL monocytes expressing CD80, PD‐L1, and PD‐L2 was significantly higher in MLWH than in MNLWH. In addition, an increased number of IM monocytes expressed CCR2, CD86, and TLR3.

At birth, a significantly higher percentage of IM monocytes expressing TLR3 was observed in HEU infants (*p* = 0.0271). At 10 weeks of age, the HEU infants had a higher level of activation in the CL monocyte subset, with a significantly increased proportion of CL monocytes expressing CCR2 (*p* = 0.0252) and CD80 (*p* = 0.0007) and a lower proportion of CL monocytes expressing TLR4 (*p* = 0.0138), compared to their HUU counterparts. While HEU infants had a reduced proportion of IM monocytes expressing CCR2 (*p* = 0.0149), no differences were observed in the expression of any markers on the IM monocytes.

A lower percentage of total NC monocytes was noted in HEU infants (*p* = 0.0406), and a lower proportion expressed CD86 (*p* = 0.0406). No differences were evident in any of the subsets or markers at 6 months of age. Additional maternal and infant monocyte data are included in Tables S16−S18 of the supplementary data.

### 3.5. Cytokine/Chemokine Profiles

All the analyses described below were performed as exploratory investigations, due to limited sample availability, to inform future functional studies with regards to markers chosen and appropriate time points to analyze. Comparisons of cytokine/chemokine data between the two cohorts of the mothers and infants are included in Tables S19, S20 of the supplementary data.

No differences were observed in the cytokine/chemokine concentrations between MLWH and MNLWH at 28 weeks’ gestation. Although not significant, slightly higher TNF‐α and IL‐6 concentrations were observed in MLWH compared to MNLWH (1.2‐fold and 1.3‐fold, respectively) while marginally lower concentrations of IL‐2 (1.5‐fold), IL‐4 (1.6‐fold), and IFN‐γ (1.1‐fold) were noted in MLWH compared to their uninfected counterparts at 28 weeks’ gestation.

Despite no significant changes, HEU infants had almost a two‐fold higher expression of IL‐8 and slightly elevated levels of IL‐4, together with lower levels of IL‐10 (1.3‐fold) and IFN‐γ at 10 weeks of age compared to the HUU infants. The slightly increased levels of IL‐4 had decreased, but the elevated IL‐8 and decreased expression of IL‐10 and IFN‐γ persisted at 6 months of age. Although not significant, the HEU infants did have notably higher concentrations of TGF‐β1 compared to the HUU cohort at 10 weeks of age which reached significance (*p* = 0.0371) at 6 months of age. While levels of TGF‐β1 decreased in both groups between 10 weeks and 6 months, these changes were not statistically significant (HUU *p* = 0.3750; HEU *p* = 0.5000).

### 3.6. Levels of CRP

Although the CRP levels of all participants were all within the normal range at all time points, the following interesting trends were noted. MLWH had higher CRP levels compared to the MNLWH. In total, 17/29 (58.6%) MLWH and 10/26 (38.5%) MNLWH had CRP levels in the highest two quartiles (*p* = 0.111). Despite not reaching significance, the levels of CRP were markedly lower at both 10 weeks and 6 months of age in the HEU cohort compared to the HUU group (8‐fold and 1.8‐fold, respectively). In total, 9/15 (60%) HUU and 3/10 (30%) HEU infants had a CRP level in the highest two quartiles, and this difference attained significance (*p* = 0.032).

The concentrations of CRP were also notably higher in both infant cohorts at 6 months of age compared to the levels detected at 10 weeks of age in the same cohorts; however, this difference was only significant in the HUU infants (*p* = 0.0273) and not in the HEU infants (*p* = 0.3125). A comparison of CRP data between mothers and infants is included in Table S21 of the supplementary data.

No significant correlations were seen between antenatal maternal CD4 counts and VLs and any of the cytokines measured.

## 4. Discussion

The lower CD4+ T‐cell count in MLWH aligns with expected HIV‐related T‐cell depletion [31]. Well‐controlled VL typically allows CD4+ T‐cell counts to exceed 500 cells/mm^3^ after several years of ART [32]. Although ART initiation dates were unknown for MLWH in this study, their median CD4+ T‐cell count was around 450 cells/mm^3^ from 22 weeks’ gestation to delivery. Pregnancy can also reduce CD4+ T‐cell counts [33], and about 11% of MLWH in the study showed advanced immunodeficiency (CD4+ T‐cell count <200 cells/mm^3^). Maternal VL records were not available for all participants, likely due to limited VL testing in the national HIV treatment program and the absence of routine point‐of‐care VL testing at the hospital during the study. Only 30% of MLWH had VLs recorded between 22 weeks’ gestation and birth, with 34% showing detectable HIV levels >1000 copies/mL. This limited data hindered detailed subgroup analysis, particularly regarding the impact of uncontrolled viral replication on small T‐cell populations.

The observed immunodeficiency and viral replication likely disrupt CD4+ T‐cell differentiation and function [34], increasing the risk of opportunistic infections and poor pregnancy outcomes [35]. It has been reported that MLWH exhibits a significantly lower CD4:CD8 ratio compared to MNLWH and that those with a ratio <0.3 at conception [33, 34] have an increased risk of preterm delivery [35]. The current study found no such associations, likely due to study design and timing of the blood tests.

At 28 weeks’ gestation, MLWH showed a significantly higher proportion of EM CD4+ T‐cells compared to MNLWH, a difference that persisted up to the time of birth.

Although an increase in CD4+ EM T‐cells (CD45RA−CCR7− and CD45RA+CCR7−) has been documented during and after pregnancy [36], this factor does not account for the elevated CD4+ EM T‐cells observed in MLWH in the current study. In contrast, Bordoni et al. [37] found a reduction in CD4+ EM T‐cells (CD45RA−CCR7−) in PLWH after initiating ART, persisting up to 24 weeks. The authors attributed this decrease to ART [37]. The discrepancy between our findings and Bordoni et al. [37] may be due to differences in ART duration and regimens. Bordoni et al. [37] studied participants on varied ART regimens, while our study involved MLWH on a consistent regimen of tenofovir disoproxil fumarate, emtricitabine, and efavirenz. The high percentage of mature CD4+ EM T‐cells in this study may stem from chronic immune activation, commonly seen in PLWH [13]. Exposure of CD4+ EM T‐cells to the pro‐inflammatory cytokine IL‐1β triggers the release of cytokines such as IL‐17 and IFN‐γ, promoting immune activation [38] and leading to T‐cell exhaustion [39].

At 28 weeks’ gestation, MLWH had significantly increased numbers of CD8+ cytotoxic T‐cells. T‐cell maturation, generally progressing from N→CM→EM1→EM2→pE1→pE2→EM4→EM3→E [29], shows maturation arrest in MLWH from the EM3 stage onwards. Although this feature has been documented in chronic HIV infection [13], it has not been observed during pregnancy. A review on HIV immunopathogenesis suggests that viral strategies prevent CD8+ T‐cells from fully maturing, impairing their viral clearance and establishing chronic disease [40]. In addition, HIV infection is associated with fewer mature CD8+ T‐cells and significantly reduced cytotoxic potential, correlating with disease progression [41, 42].

Interestingly, HEU infants also exhibited disrupted CD4+ EM T‐cell maturation, with higher CD4+ EM1 and lower CD4+ EM4 percentages at birth, persisting at 10 weeks of age. This reduction aligns with findings that have been reported by Clerici et al. [43] and suggests impaired infection clearance due to incomplete T‐cell activation, hindering the pro‐inflammatory cytokine production that is vital for an effective response to infection [44]. Similarly, a study investigating the stereotypic expansion of Tregs and T‐helper (Th)17 cells reports disrupted T‐cell maturation preceded by limited T‐cell receptor diversity in memory T‐cells and lower differentiated Th1, Th2, and Th17 CD4+ T‐cell percentages [6].

Chronic immune activation is a key feature of HIV infection [13, 45]. This is evident from the assessment of activation markers in MLWH at 28 weeks’ gestation and at the time of birth.

Increased PD‐1 expression in MLWH at 28 weeks’ gestation and at the time of birth suggests greater antigen exposure [29]. Elevated PD‐1 expression is found in highly activated T‐cells [46] and during chronic inflammation, while persistent PD‐1 expression has been linked to T‐cell exhaustion [46]. Binding of PD‐1 to PD‐L1 or PD‐L2 prevents CD28 activation, reducing T‐cell proliferation [46]. Upregulation of PD‐1 on HIV‐specific CD4+ and CD8+ T‐cells, along with the interaction between PD‐1 and PD‐L1, leads to T‐cell dysfunction and disease progression [47]. PD‐1 expression on T‐cells rises during healthy pregnancy, while PD‐L1 expression on monocytes remains similar in pregnant and non‐pregnant women [48]. Despite the antigen‐experienced environment and activated immune state, fewer IM of MLWH expressed PD‐L1 at 28 weeks’ gestation. This study observed ongoing T‐cell activation and proliferation in MLWH at birth. To our knowledge, this is the first report of increased PD‐1 expression on T‐cells in PWLWH.

No significant differences were observed in PD‐1 expression on CD4+ and CD8+ T‐cells between HEU and HUU infants at birth. At 10 weeks of age; however, HEU infants had increased PD‐1 expression on TEMRA pE1 CD4+ T‐cells. This activation was temporary and no longer evident at 6 months of age, with a reduced proportion of CD4+ EM and CD8+ TEMRA E T‐cells expressing PD‐1 in HEU compared to HUU infants observed at this time point. These findings align with a study by Miles et al. [49], which found increased PD‐1 expression on fully differentiated CD4+ T‐cells of HEU infants at 10 weeks of age. However, a Malawian study reported no difference in PD‐1 expression on T‐cells between HEU and HUU infants at birth or 5 to 9 weeks of age [50]. The discrepancy may be due to the timing of the analysis.

As was the case with the mothers, HEU infants exhibited lower PD‐L1 expression at birth, specifically on CL monocytes, but not on IM and NC monocytes. This difference was no longer evident by 10 weeks of age, at which point HEU infants showed increased activation in T‐cell and monocyte subsets, indicated by a higher number of CD8+ T‐cells expressing PD‐1 and a greater percentage of CL monocytes expressing CCR2 and CD80. These changes were transient and not observed at later time points.

The current study found increased PD‐1 and CD57 expression in CD4+EM T‐cells at 28 weeks’ gestation and at birth, indicating highly activated/exhausted and senescent T‐cells.

The exhausted T‐cell phenotype, initially characterized by co‐expression of inhibitory molecules such as PD‐1, cytotoxic T‐lymphocyte‐associated protein 4 (CTLA‐4), T cell immunoglobulin and mucin domain 3 (TIM‐3), lymphocyte‐activation gene 3 (LAG‐3) and T cell immunoreceptor with Ig and ITIM domains (TIGIT), exhibits impaired cytotoxicity and reduced IL‐2 production [51]. Additional markers such as CD39, CD69, and CD73 may further define exhausted T‐cells [51].

A review on T‐cell exhaustion and epigenetics proposes that exhausted T‐cells could be categorized into precursor and terminally exhausted phenotypes based on transcriptional characteristics like T‐cell factor‐1 [51]. The authors recommend integrating inhibitory marker expression, functional studies, and transcriptional characteristics to define exhausted T‐cells comprehensively [51]. Increased CD57 expression on cytotoxic CD4+ T‐cells correlates with the cytotoxicity of highly differentiated senescent T‐cells [51, 52].

MLWH exhibited a markedly lower CD4:CD8 ratio than MNLWH [53, 54]. PLWH with low CD4:CD8 ratios, despite effective ART, experience increased immune senescence and inflammation [54]. This has been associated with inadequate control of chronic latent coinfections like cytomegalovirus and Epstein‐Barr virus [54]. Increased CD57 expression in CD4+ and CD8+ EM T‐cells at 28 weeks’ gestation suggests T‐cell exhaustion in MLWH. This exhaustion reduces T‐cell proliferation and cytotoxic capacity, indicated by lower levels of IFN‐γ, IL‐2, and TNF‐α, impairing immune responses to viral infections. Consequently, this may affect HIV control in MLWH and increase susceptibility to viral infection in HEU infants during their first 10 weeks of life.

HEU infants exhibited an increased proportion of CD8+ T‐cells, including CD8+ EM (specifically EM1) and TEMRA subtype E T‐cells, expressing CD57 at birth. This increase persisted in CD8+ EM1 T‐cells at 10 weeks of age, with more CD8+ TEMRA subtype E T‐cells expressing CD57. Conversely, at 6 months of age, HEU infants had fewer CD4+ and CD8+ T‐cells expressing CD57. Persistent antigen epitope recognition is necessary for increased PD‐1 expression on CD8+ T‐cells, though studies on this have shown mixed results [50]. The short‐lived T‐cell activation and exhaustion observed in the HEU infants may be a persistent response to antigen exposure in utero from which they recover 6 months post exposure.

Regulatory T‐cells are essential for balancing pro‐ and anti‐inflammatory responses and maintaining immune tolerance at the maternal‐fetal interface during pregnancy, ensuring fetal growth [55, 56].

At 28 weeks’ gestation, MLWH had a higher percentage of total Tregs than MNLWH, with no significant differences in CD39, CD45RA, and Helios expression. Increased numbers of Tregs have been reported in chronic HIV infection, exacerbating T‐cell exhaustion by enhancing IL‐10 synthesis, which suppressed T‐cell proliferation [57]. The findings of the current study indicate a notably altered maternal T‐cell environment, marked by CD4+, CD8+, and Treg cell exhaustion.

The initial wave of Tregs in neonates is crucial for lifelong immune health, particularly in preventing autoimmunity and allergies [58, 59]. Although not statistically significant, the present study noted a lower percentage of Tregs in HEU infants. We hypothesize that this could be due to cell migration to mitigate gut epithelial damage [60]. Intestinal damage and an altered gut microbiome have been documented both in PLWH [61, 62] and HEU infants [63]. Reduced Tregs in preterm newborn infants are linked to a higher risk of necrotizing enterocolitis [64], underscoring the critical relationship between gut integrity and Tregs. Future research should therefore focus on gut health.

We further hypothesize that the lower Tregs observed in HEU infants may involve type 1 Tregs (Tr1), which transiently express FoxP3 and immune checkpoint molecules like PD‐1, CTLA‐4, and TIM‐3, regulating T‐cell proliferation via TGF‐β1 and IL‐10 [65]. This could explain the increased levels of T‐cell activation and exhaustion observed in the HEU infants. Since the current panel could not reliably detect Tr1 Tregs, further research is necessary. We also observed that, based on CD45RA expression, HEU infants appeared to have a more immature subset of Tregs. To explore this further, future studies should use a broader panel of maturation markers, including CCR7, CD27, CD28, and CD45RA [29].

Both infant cohorts exhibited a significant proportion of thymus‐derived Tregs (tTregs) with elevated Helios expression, which is correlated with increased cytolytic activity, at 10 weeks of age [66]. Stadinski et al. [67] identified that neonatal peptidyl arginine deiminase type IV‐specific thymocytes are exported and sustained in peripheral circulation during the neonatal period, with a potential increase during inflammatory conditions. The absence of data confirming or refuting these observations in HEU infants in the existing literature indicates that the present study is pioneering in its phenotypic characterization of peripheral Tregs (pTregs) and tTregs in HEU infants.

The elevated percentage of tTregs observed in MLWH at 28 weeks’ gestation, at birth, and in both infant groups at 10 weeks of age suggests enhanced cytolytic activity, accompanied by increased production of granzymes and perforin [66].

The observed increase in CD39+ Tregs in MLWH at 28 weeks of gestation, which becomes statistically significant at birth, indicates a potential metabolic disruption characterized by impaired degradation of adenosine triphosphate (ATP) to adenosine monophosphate [68]. The expression of CD39, which facilitates ATP degradation, exhibited reduced disruption in HEU infants at birth, persisting up to 10 weeks of age, with an increase noted at 6 months [68]. Given the critical role of Tregs in maintaining immune homeostasis, any functional alterations could have substantial implications for infant health, thereby necessitating further investigation.

No significant differences were observed in the total percentage of CL or NC monocytes between MLWH and MNLWH at 28 weeks’ gestation. However, MLWH exhibited an increased total percentage of IM monocytes, which persisted until the time of birth. In vitro experiments suggest that Tregs co‐cultured with monocytes induce an alternatively activated state in the latter, characterized by diminished expression of activation markers and limited secretion of pro‐inflammatory cytokines in response to lipopolysaccharide (LPS) [69]. These findings imply that elevated levels of Tregs and IM monocytes may mitigate the pro‐inflammatory environment in MLWH [70], as previously documented [13, 45]. At birth, MLWH also demonstrated a reduced total percentage of CL monocytes, which, in conjunction with impaired LPS responses, could compromise their ability to respond to pathogens [70].

At birth and at 6 months of age, no significant differences were observed in the total percentage of CL, IM, and NC monocytes between HEU and HUU infants. However, at 10 weeks of age, HEU infants exhibited a lower percentage of total NC monocytes, indicating a potential limitation in patrolling function, tissue repair, and dead cell removal, which may lead to increased inflammation [71].

A longitudinal analysis of peripheral blood monocytes during early, mid, and late gestation, utilizing mass cytometry and ribonucleic acid (RNA) sequencing, demonstrated a shift from CL to IM/NC monocytes as pregnancy progresses [72]. In the current study, an increased total percentage of IM monocytes was observed in MLWH at 28 weeks’ gestation which persisted at the time of birth. In addition, a higher immune activation state, as seen in MLWH T‐cell subsets, was also present in the monocyte subsets.

At 28 weeks’ gestation, MLWH had increased CL monocytes expressing CCR2, a higher percentage of IM monocytes expressing CCR2, CD86, and TLR3, and more NC monocytes expressing CD80. However, PD‐L1 and PD‐L2 expression was lower in IM and NC monocytes, respectively. These findings suggest activation due to persistent antigen exposure, potentially contributing to inflammation.

Studies in MNLWH have linked mid‐pregnancy inflammation with adverse birth outcomes like preterm birth and preeclampsia [73], while studies in MLWH have specifically associated monocyte activation with adverse outcomes [74, 75]. The timing and extent of such inflammation are crucial, as some immune activation is necessary for normal pregnancy establishment and maintenance. The Prematurity Immunology in HIV‐infected Mothers and their Infants Study (PIMS) in Cape Town, RSA, found that low immune activation with predominantly anti‐inflammatory monocytes was linked to preterm birth in women starting ART during pregnancy [76]. At the time of birth, MLWH had a different monocyte ratio (proportion of CL over IM and NC populations) compared to their profile at 28 weeks’ gestation and MNLWH at the same time. This was due to a lower number of CL monocytes and a higher number of IM monocytes. In addition, CL monocytes had higher CD80, PD‐L1, and PD‐L2 expression, while IM monocytes showed increased CCR2, CD86, and TLR3 expression, indicating CL monocyte exhaustion and IM monocyte activation. Despite these activation differences, there were no differences in adverse birth outcomes between MLWH and MNLWH.

A significantly elevated proportion of IM monocytes expressing TLR3 was identified in HEU infants at birth. The expression of TLR3 is associated with the recognition of viral RNA [77], suggesting that the heightened TLR3 expression and transient activation state in HEU infants may be a response to in utero HIV exposure [78]. Furthermore, HEU infants exhibited a reduced number of CL monocytes expressing TLR4 at 10 weeks of age. TLR4, present on antigen‐presenting cells, recognizes LPS associated with Gram‐negative bacteria [11], and a decrease could imply a diminished capacity to recognize Gram‐negative bacteria, which may increase the risk of infection.

In contrast to MLWH, HEU infants demonstrated a decreased proportion of NC monocytes expressing CD86 at 10 weeks of age, indicating a potentially compromised antigen‐presenting capability [79].

Dirajlal‐Fargo et al. [80] reported increased monocyte activation and inflammation in MLWH and HEU infants at birth, corroborating the elevated levels of CCR2‐expressing CL monocytes in MLWH at 28 weeks’ gestation and in HEU infants at 10 weeks of age.

The reduced proportion of CCR2+ IM monocytes in HEU infants at 10 weeks of age suggests impaired chemotaxis of these cells, which could potentially impair their ability to invade into tissue and subsequently differentiate into macrophages [70].

Research indicates that monocytes are progressively activated during pregnancy, evidenced by heightened responsiveness to stimuli like LPS and elevated production of antiviral and pro‐inflammatory cytokines such as IL‐6 [81]. This activation is essential for maintaining pregnancy, and immune anergy to TLR stimulation has been linked to preterm birth in women starting ART during pregnancy [76]. While no significant differences were observed in maternal cytokine/chemokine levels, MLWH exhibited higher IL‐6 and TNF‐α and lower IFN‐γ and IL‐2 concentrations. The increased IL‐6 and TNF‐α might be due to type 1 IFN‐α production by plasmacytoid dendritic cells in response to viral exposure, indicative of hyperactivated innate inflammation [82]. Production of IFN‐α increases during chronic immune activation due to HIV‐1 infection, potentially causing apoptosis of uninfected CD4+ T‐cells and contributing to immune exhaustion [40]. In contrast, lower levels of IFN‐γ have been reported in MLWH, presumably secondary to reduced T‐helper 1 type responses, a classic feature of HIV immunobiology [83].

Naïve CD4+ T‐cells differentiate into Tregs in the presence of TGF‐β1 [84]. Although TGF‐β1 levels were higher in HEU infants at 10 weeks and 6 months of age, it did not markedly impact Treg proportions [84]. Slightly increased IL‐4 levels in HEU might indicate a stronger Th2 response [52]. However, decreased IL‐10 levels in HEU infants compared to HUU suggest a limited ability to suppress pro‐inflammatory responses [85]. Disrupted CD4+ T‐cell maturation observed in HEU infants at birth persisted at 10 weeks of age, indicating potential impaired antigen recognition. Despite this, HEU infants exhibited higher levels of neutrophil chemotactic factor, IL‐8, which increased over time, and increased activation markers on monocytes/macrophages at 10 weeks of age, suggesting the innate immune system might compensate for impaired T‐cell differentiation. Overall, the altered cytokine milieu might point to altered immune programing in HEU infants. All cytokine/chemokine analyses were performed as exploratory analyses to inform future functional studies with regards to markers chosen and appropriate time points to analyze.

Further research is needed to explore possible mechanistic pathways.

Higher CRP levels in MLWH compared to the uninfected cohort suggest a more pro‐inflammatory state, aligning with increased activation and exhaustion in the T‐cell subset. In contrast to their mothers, HEU infants had lower CRP levels compared to the HUU cohort. This finding differs from data reported by Prendergast et al. [86] who found significantly higher CRP levels in HEU infants at 6 weeks and 6 months of age. The Prendergast study was conducted in the pre‐ART era and prior to the introduction of cotrimoxazole prophylaxis for all HEU infants. While the effect of ART on infant inflammation cannot be excluded, it is more likely that cotrimoxazole might have altered the microbiome of HEU infants or reduced their overall microbial burden and translocation of bacterial products, thereby limiting systemic IL‐6‐mediated acute phase responses.

The current study found that HEU infants exhibited suboptimal growth from birth to 12 months of age compared to HUU infants. Elevated levels of the sCD14 were observed in MLWH compared to MNLWH and has also been reported by others [45]. In addition, a study from India supports this association, linking elevated sCD14 with low LAZ and WAZ in HEU infants at birth [87]. Persistent suboptimal growth in HEU infants indicates additional contributing factors. For instance, reduced β‐hydroxybutyric acid levels in MLWH at 28 weeks’ gestation and HEU infants at 6 months [8], along with decreased breastfeeding between 6 and 12 months [9], previously reported from the Siyakhula study, may have contributed to these poor growth parameters.

A major strength of this study is its integration within the larger multi‐disciplinary Siyakhula study allowing for the identification of associations between immunological changes and clinical outcomes, such as infant growth and metabolic aberrations. Additionally, results were adjusted for gestational age, determined by early ultrasound, a critical factor in neonatal research. All MLWH participants received the same ART regimen from 22 weeks’ gestation and throughout the study. However, a limitation of the study is the fluctuation of participant numbers at the various time points for reasons mentioned previously.

Researchers are gradually deciphering the immunological changes in HEU infants, but many questions remain. This study’s findings require validation in larger longitudinal cohorts.

Additionally, a future study assessing differences between infants lost to follow‐up and those continuously monitored, with the help of ward‐based community outreach teams, could provide insights into the morbidity and mortality of those participants lost to follow‐up. Future T‐cell studies with an expanded panel of co‐stimulatory and co‐inhibitory receptors may further elucidate the roles of these biomarkers in T‐cell exhaustion [78]. Future work should also improve the phenotypic characterization of Tregs by, for instance, including markers such as CD127, TIGIT, and Neuropilin 1 [60, 88, 89].

A significant area lacking comprehensive understanding is the long‐term impact of these immunological changes as HEU children progress beyond infancy. Additionally, the impact of maternal ART on immune activation in HEU children is an important consideration needing further study. Notably, the specific roles of maternal immunization and timing of ART initiation could be key to understanding and potentially mitigating the risks of infections [90].

## 5. Conclusion

The findings of this study suggest that increased maternal immune activation affects T‐cell maturation, activation, and regulation, as well as monocyte activation and responsiveness in HEU infants at birth and 10 weeks of age, which could result in increased susceptibility to infection during this period. Supportive measures may include maternal vaccination, more frequent clinic visits, and possibly, a supplemented diet for mothers during exclusive breastfeeding.

Although the immune system of HEU infants seems to recover by 6 months of age, it remains uncertain whether complications might surface later, given the critical role of immune ontogeny in lifelong immune homeostasis. Many questions remain regarding the persistence and full impact of these changes, as well as the underlying mechanisms driving these alterations. Further longitudinal research focusing on the long‐term immune function of HEU children, and the multifaceted effects of maternal health and treatment regimens is necessary to address these gaps.

## Funding

This work was supported by the National Research Foundation Thuthuka grant (121857), International AIDS Society CIPHER grant (217/560‐FEU), and the National Health Laboratory Service Research Trust Grant (94669).

## Disclosure

An earlier version of this content has been presented as a thesis [91].

## Conflicts of Interest

The authors declare no conflicts of interest.

## Supporting Information

Additional supporting information can be found online in the Supporting Information section.

## Supporting information


**Supporting Information** Figure S1. Examples of flow cytometry data analysis including A: Basic data clean‐up gating strategy; B: UMAP and CITRUS analysis results; C: Infant T‐cell t‐SNEs depicting Regulatory T‐cell clusters expressing CD25, FoxP3 or Helios and D: Examples of t‐SNE gating of the monocyte subset. Table S1. Infant anthropometric measurements and Z‐scores at 10 weeks, 6 months and 12 months of age. Table S2. Comparison of CD4+ T‐cell maturation stages between mothers living with and without HIV at 28 weeks’ gestation and at the time of birth. Table S3. Comparison of CD4+ T‐cell maturation stages between HIV‐exposed and unexposed infants at birth, ten weeks and at six months age. Table S4. Comparison of CD8+ T‐cell maturation stages between mothers living with and without HIV at 28 weeks’ gestation and at the time of birth. Table S5. Comparison of CD8+ T‐cell maturation stages between HIV‐exposed and unexposed infants at birth, 10 weeks and 6 months of age. Table S6. Percentage of PD‐1 expression on CD4+ T‐cells between mothers living with and without HIV at 28 weeks’ gestation and the time of birth. Table S7. Percentage of PD‐1 expression on CD4+ T‐cells between HIV‐exposed and unexposed infants at birth, 10 weeks and 6 months of age. Table S8. Percentage of CD57 expression on CD4+ T‐cells between mothers living with and without HIV at 28 weeks’ gestation. Table S9. Percentage of CD57 expression on CD4+ T‐cells HIV‐exposed and unexposed infants at birth, 10 weeks and 6 months of age. Table S10. Percentage of PD‐1 expression on CD8+ T‐cells between mothers living with and without HIV at 28 weeks’ gestation and the time of birth. Table S11. Percentage of PD‐1 expression on CD8+ T‐cells between HIV‐exposed and unexposed infants at birth, 10 weeks and 6 months of age. Table S12. Percentage of CD57 expression on CD8+ T‐cells between mothers living with and without HIV at 28 weeks’ gestation. Table S13. Percentage of CD57 expression on CD8+ T‐cells between HIV‐exposed and unexposed infants at birth, 10 weeks and 6 months of age. Table S14. Percentage of regulatory T‐cell marker expression on CD4+T‐cells between mothers living with and without HIV at the time of birth. Table S15. Percentage of regulatory T‐cell marker expression on CD4+T‐cells between HIV‐exposed and unexposed infants from birth to 6 months of age. Table S16. Percentage of monocyte marker expression between mothers living with and without HIV at 28 weeks’ gestation and at the time of birth. Table S17. Percentage of monocyte marker expression between HIV‐exposed and unexposed infants at birth and 10 weeks of age. Table S18. Percentage of monocyte marker expression between HIV‐exposed and unexposed infants at 6 months of age. Table S19. Comparison of the cytokine/chemokine data between the two cohorts of mothers at 28 weeks’ gestation. Table S20. Comparison of cytokine/chemokine data between the two cohorts of infants at 10 weeks and 6 months of age. Table S21. Comparison of C‐reactive protein data between mothers and infants.

## Data Availability

The data that support the findings of this study are available from the corresponding author upon reasonable request.
