# Supplementary material for: The Effects of In Utero HIV and Antiretroviral Therapy Exposure on Infant T‐Cell and Monocyte Activation, Function, and Regulation of Immune‐Modulatory Pathways
Source: Mediators Inflamm. 2026 Mar 24;2026:2928164. doi: 10.1155/mi/2928164 (PMC13140189; doi:10.1155/mi/2928164)
Supplement: Supplementary file 1 — Supporting Information Figure S1. Examples of flow cytometry data analysis including A: Basic data clean‐up gating strategy; B: UMAP and CITRUS analysis results; C: Infant T‐cell t‐SNEs depicting Regulatory T‐cell clusters expressing CD25, FoxP3 or Helios and D: Examples of t‐SNE gating of the monocyte subset. Table S1. Infant anthropometric measurements and Z‐scores at 10 weeks, 6 months and 12 months of age. Table S2. Comparison of CD4+ T‐cell maturation stages between mothers living with and without HIV at 28 weeks’ gestation and at the time of birth. Table S3. Comparison of CD4+ T‐cell maturation stages between HIV‐exposed and unexposed infants at birth, ten weeks and at six months age. Table S4. Comparison of CD8+ T‐cell maturation stages between mothers living with and without HIV at 28 weeks’ gestation and at the time of birth. Table S5. Comparison of CD8+ T‐cell maturation stages between HIV‐exposed and unexposed infants at birth, 10 weeks and 6 months of age. Table S6. Percentage of PD‐1 expression on CD4+ T‐cells between mothers living with and without HIV at 28 weeks’ gestation and the time of birth. Table S7. Percentage of PD‐1 expression on CD4+ T‐cells between HIV‐exposed and unexposed infants at birth, 10 weeks and 6 months of age. Table S8. Percentage of CD57 expression on CD4+ T‐cells between mothers living with and without HIV at 28 weeks’ gestation. Table S9. Percentage of CD57 expression on CD4+ T‐cells HIV‐exposed and unexposed infants at birth, 10 weeks and 6 months of age. Table S10. Percentage of PD‐1 expression on CD8+ T‐cells between mothers living with and without HIV at 28 weeks’ gestation and the time of birth. Table S11. Percentage of PD‐1 expression on CD8+ T‐cells between HIV‐exposed and unexposed infants at birth, 10 weeks and 6 months of age. Table S12. Percentage of CD57 expression on CD8+ T‐cells between mothers living with and without HIV at 28 weeks’ gestation. Table S13. Percentage of CD57 expression on CD8+ T‐cells between HIV [file MI-2026-2928164-s001.docx]

***
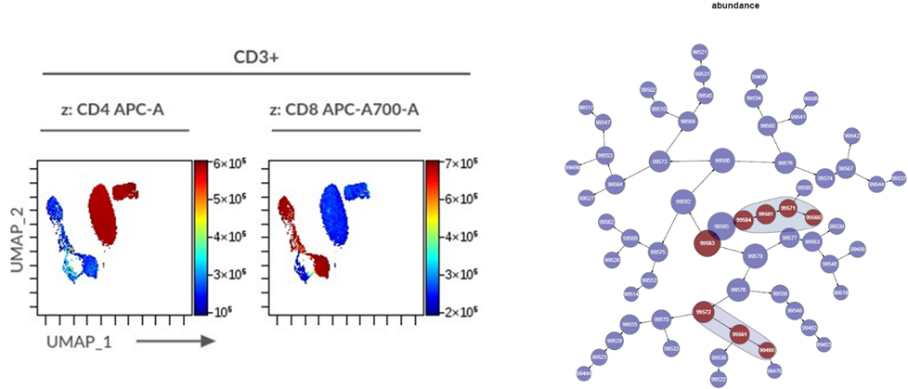
*A B**


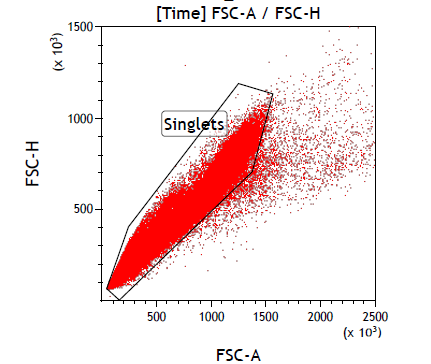

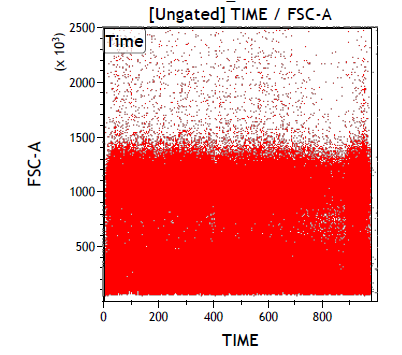

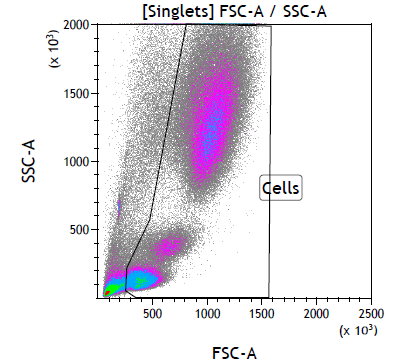


**
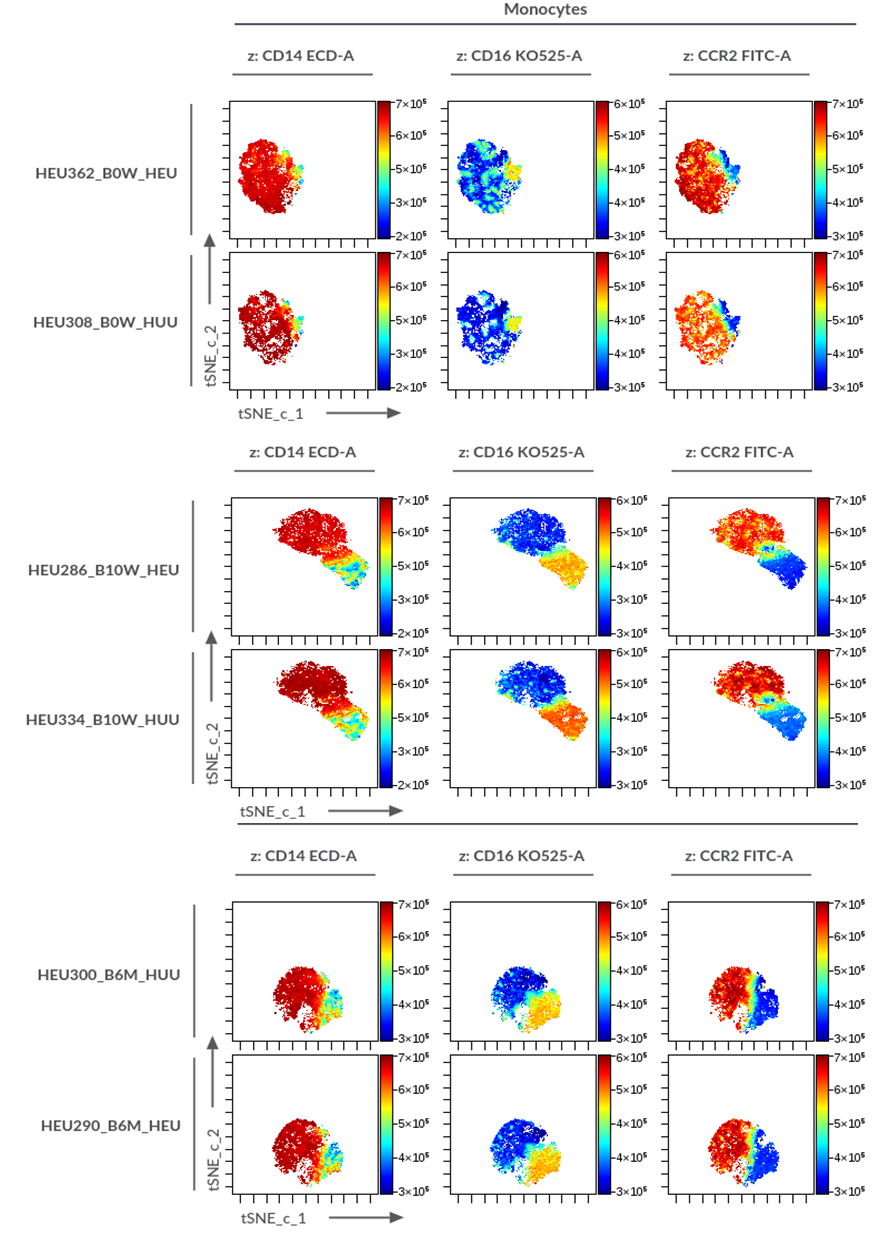

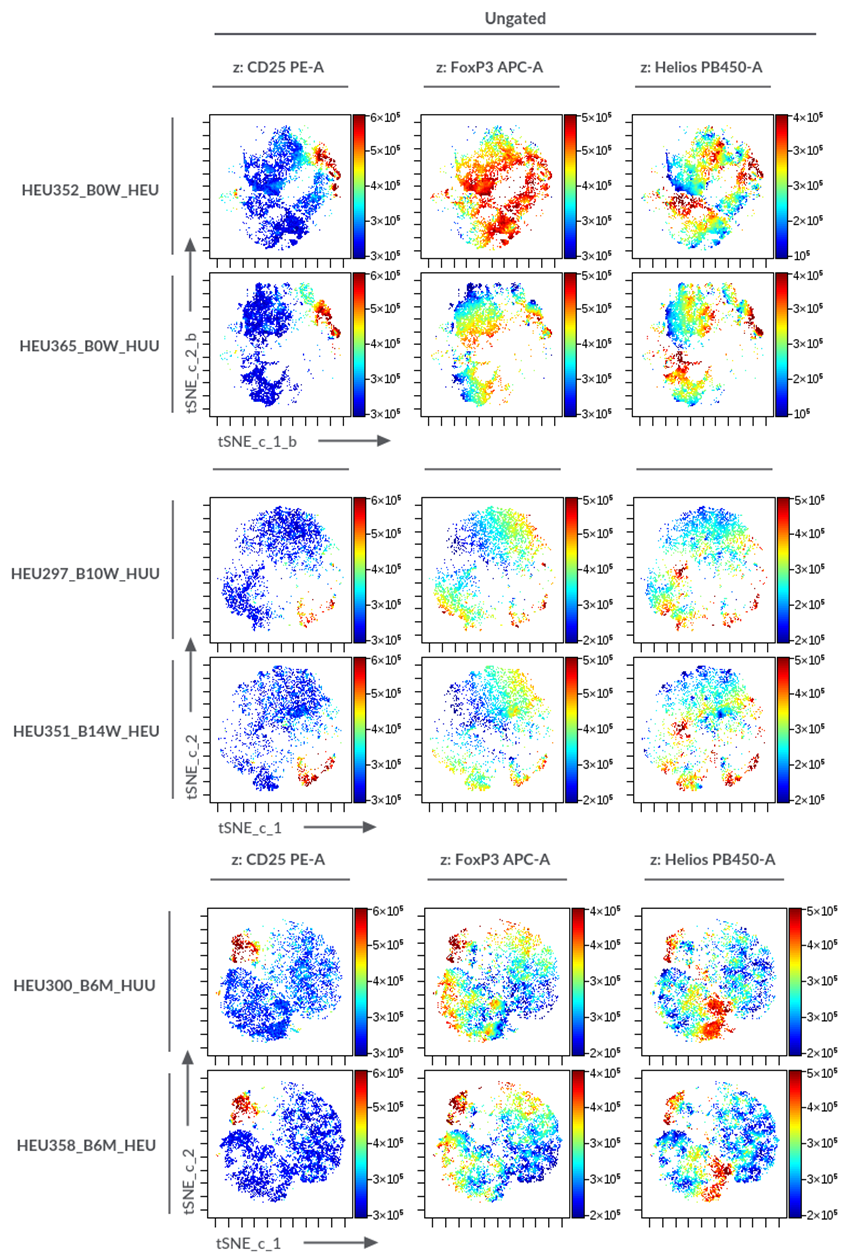
C D**

***Figure S1: Examples of flow cytometry data analysis including A: Basic data clean-up gating strategy; B:* UMAP and CITRUS analysis results; C: *Infant T-cell t-SNEs depicting Regulatory T-cell clusters expressing CD25, FoxP3 or Helios and D:* Examples of t-SNE gating of the monocyte subset**

Abbreviations: A=Alexa Fluor/Area; APC=Allophycocyanin; B0W=Babies at birth; B10W=Babies at ten weeks of age; B6M=Babies at six months of age; c=Channel; CCR=C-C motif chemokine receptor; CD=Cluster of differentiation; ECD=R-PE-Texas-red; FITC=Fluorescein isothiocyanate; FSC=Forward scatter; HEU=HIV-exposed-uninfected; HIV=Human immunodeficiency virus; HUU=HIV-unexposed-uninfected; IDs=identities; KO=Krome orange; PB=Pacific blue; PE=Phycoerythrin; SSC=Side scatter; tSNE=t-distributed Stochastic Neighbour Embedding; z=Z-channel

**Table S1: Infant anthropometric measurements and Z-scores at ten weeks, six months and 12 months of age**

|  | **Ten Weeks** | | | **Six Months** | | | **12 Months** | | |
| --- | --- | --- | --- | --- | --- | --- | --- | --- | --- |
| **Variable** | **HEU (n=70)** | **HUU (n=71)** | **p-Value** | **HEU (n=70)** | **HUU (n=71)** | **p-Value** | **HEU (n=70)** | **HUU (n=71)** | **p-Value** |
| **Weight (g)** | 5200 (±700) | 5500 (±800) | **0.0290** | 7100 (±800) | 7600 (±800) | **0.0051** | 9000 (±1100) | 9400 (±1300) | 0.2456 |
| **Length (cm)** | 56.9 (±2.4) | 57.1 (±2.9) | 0.7190 | 65.4 (±3.1) | 66.1 (±2.7) | 0.1962 | 74.5 (±3.1) | 73.9 (±2.6) | 0.2313 |
| **Weight-for-length Z-score** | 0.18 (±1.76) | 0.68 (±1.39) | 0.1670 | -0.22 (±0.88) | 0.31 (±0.95) | **0.0156** | -0.5 (-1.1 – 0.3)* | 0.2 (-0.5 – 1.0)* | **0.0227** |
| **Weight -for-age Z-score** | -0.35 (±1.35) | 0.14 (±1.18) | 0.1986 | -0.36 (±0.93) | 0.11 (±1.02) | **0.0369** | -0.56 (±0.93) | -0.11 (±1.28) | **0.0408** |
| **Length-for-age Z-score** | -0.48 (±1.38) | -0.31 (±1.53) | 0.5648 | -0.22 (±1.46) | -0.04 (±1.37) | 0.5700 | -0.68 (±1.15) | -0.59 (±1.43) | 0.7367 |
| **BMI-for-age Z-score** | -0.08 (±1.57) | 0.49 (±1.19) | **0.0366** | -0.29 (±0.92) | 1.19 (±1.00) | **0.0339** | -0.23 (±1.12) | 0.34 (±1.25) | **0.0262** |
| **HC (cm)** | 39.5 (±1.7) | 39.9 (±1.6) | 0.2238 | 43.3 (±1.5) | 44.0 (±1.4) | **0.0161** | 46.4 (±1.5) | 46.8 (±1.4) | 0.2805 |
| **HC Z-score** | 0.66 (±1.65) | 0.98 (±1.05) | 0.4995 | 0.78 (1.33) | 1.24 (±1.16) | 0.1147 | 0.39 (±1.00) | 0.96 (±1.06) | **0.0420** |
| **MUAC (cm)** | 13.7 (±1.0) | 14.2 (±1.4) | 0.1989 | 14.5 (±1.1) | 15.1 (±1.0) | **0.0116** | 15.4 (±1.2) | 15.9 (±1.4) | 0.1323 |
| **MUAC Z-score** | Not calculated | | | 0.40 (±1.05) | 0.97 (±0.90) | **0.0230** | 0.76 (±0.95) | 1.17 (±1.05) | 0.1096 |

Abbreviations: BMI=Body mass index; cm=Centimetres; g=Grams; HC=Head circumference; HEU=HIV-exposed-uninfected; HUU=HIV-unexposed-uninfected; MUAC=Mid-upper arm circumference

P-values denoted in bold indicate significance (p<0.05)

Results are presented as mean and standard deviation, except where indicated by an asterisk (*) in which case the results are presented as mean and standard deviation.

**Table S2: Comparison of CD4+ T-cell maturation stages between mothers living with and without HIV at 28 weeks' gestation and at the time of birth**

|  | **Mothers at 28 Weeks’ gestation** | | | **Mothers at the time birth** | | |
| --- | --- | --- | --- | --- | --- | --- |
| **Description** | **MLWH (n=42)** | **MNLWH (n=33)** | **p-Value** | **MLWH (n=33)** | **MNLWH (n=31)** | **p-Value** |
| Total % CD4+ T-cells | 55.8 (41.3 – 68.2) | 71.7 (63.0 – 77.4) | **0.0001** | 53.2 (38.8 – 62.3) | 66.3 (58.5 – 73.0) | **0.0004** |
| CD4 Naïve | 27.3 (19.3 – 37.3) | 32.9 (22.3 – 42.9) | 0.2351 | 30.0 (19.2 – 42.3) | 32.1 (24.5 – 45.4) | 0.2563 |
| CD4 Central Memory | 49.9 (42.4 – 53.9) | 48.5 (42.7 – 57.7) | 0.9829 | 33.2 (27.3 – 39.3) | 37.1 (34.6 – 39.9) | **0.0488** |
| CD4 EM | 14.4 (11.3 – 22.0) | 13.0 (10.4 – 16.7) | **0.0437** | 10.7 (7.1 – 16.3) | 8.9 (4.9 – 11.6) | **0.0355** |
| CD4 EM1 | 31.0 (24.6 – 42.9) | 49.8 (33.6 – 55.4) | **0.0008** | 23.9 (16.4 – 34.9) | 40.0 (25.5 – 51.1) | **0.0022** |
| CD4 EM2 | 0.44 (0.29 – 0.86) | 0.37 (0.14 – 0.65) | 0.1454 | 1.0 (0.7 – 1.5) | 0.8 (0.7 – 1.5) | 0.6898 |
| CD4 EM3 | 36.2 (25.4 – 51.5) | 21.2 (10.3 – 40.9) | **0. 0117** | 41.2 (29.0 – 55.4) | 27.7 (12.0 – 45.7) | **0.0071** |
| CD4 EM4 | 27.2 (19.0 – 34.6) | 27.6 (22.9 – 32.9) | 0.6405 | 25.4 (19.0 – 37.4) | 29.0 (24.8 – 35.2) | 0.1825 |
| CD4 TEMRA | 0.08 (0.04 – 0.20) | 0.13 (0.03 – 0.28) | 0.5686 | 13.9 (11.1 – 20.0) | 15.0 (12.6 – 21.8) | 0.7006 |
| CD4 E | 61.9 (29.2 – 95.0) | 68.0 (21.5 – 95.9) | 0.8325 | 95.6 (93.2 – 97.7) | 95.9 (93.8 – 97.6) | 0.8081 |
| CD4 pE1 | 10.0 (0.6 – 33.3) | 1.0 (0.0 – 16.7) | **0.0330** | 0.3 (0.1 – 0.4) | 0.4 (0.2 – 0.5) | 0.3369 |
| CD4 pE2 | 6.2 (0.9 – 26.8) | 2.4 (0.0 – 20.7) | 0.1793 | 1.7 (0.8 – 3.7) | 2.3 (1.3 – 3.3) | 0.3775 |

Abbreviations: CD=Cluster of differentiation; EM=Effector memory; MLWH=Mothers living with HIV; MNLWH=Mothers not living with HIV; TEMRA=Terminally differentiated effector memory cells re-expressing CD45RA: E (CD27-CD28-), pE1 (CD27+CD28+), pE2 (CD27+CD28-)

P-Values denoted in bold indicate significance (p<0.05)

Results are presented as median and interquartile range.

**Table S3: Comparison of CD4+ T-cell maturation stages between HIV-exposed and unexposed infants at birth, ten weeks and at six months age**

|  | **Infants at the time of birth** | | | **Infants at ten weeks of age** | | | **Infants at six months age** | | |
| --- | --- | --- | --- | --- | --- | --- | --- | --- | --- |
| **Description** | **HEU (n=28)** | **HUU (n=26)** | **p-Value** | **HEU (n=16)** | **HUU (n=15)** | **p-Value** | **HEU (n=10)** | **HUU (n=15)** | **p-Value** |
| Total % CD4+ T-cells | 75.2 (68.5 – 81.0) | 73.8 (65.5 – 83.8) | 0.6526 | 78.3 (68.1 – 83.1) | 75.2 (68.6 – 85.3) | 0.8744 | 72.0 (69.5 – 75.4) | 65.8 (56.8 – 82.2) | 0.5791 |
| CD4 Naïve | 48.8 (35.9 – 61.0) | 35.5 (30.4 – 51.1) | **0.0476** | 65.3 (30.9 – 73.4) | 46.6 (31.7 – 63.5) | 0.4292 | 73.9 (69.0 – 80.2) | 67.6 (61.2 – 75.7) | 0.1138 |
| CD4 Central Memory | 44.9 (34.1 – 62.3) | 59.6 (35.9 – 63.5) | 0.2882 | 28.9 (20.8 – 64.2) | 39.3 (31.1 – 59.6) | 0.5271 | 23.2 (18.5 – 30.2) | 24.0 (19.1 – 31.5) | 0.6338 |
| CD4 EM | 2.8 (2.1 – 3.5) | 2.3 (1.5 – 3.8) | 0.3848 | 4.9 (3.8 – 5.6) | 4.0 (3.5 – 5.5) | 0.6295 | 2.9 (2.2 – 3.7) | 3.7 (3.2 – 5.0) | **0.0437** |
| CD4 EM1 | 96.4 (95.1 – 97.3) | 94.3 (87.5 – 97.1) | **0.0376** | 85.9 (75.3 – 88.6) | 76.2 (66.2 – 86.5) | **0.0316** | 52.2 (51.0 – 65.1) | 55.7 (51.3 – 70.3) | 0.4388 |
| CD4 EM2 | 2.6 (2.0 – 4.1) | 4.0 (2.1 – 8.1) | 0.1309 | 2.5 (1.3 – 4.5) | 2.4 (0.9 – 5.2) | 0.9999 | 28.3 (24.6 – 38.7) | 26.9 (19.4 – 40.1) | 0.5087 |
| CD4 EM3 | None Detected | | | 1.1 (0.2 – 8.8) | 3.1 (0.1 – 17.6) | 0.5143 | 1.6 (0.8 – 2.0) | 2.8 (1.2 – 3.8) | 0.0280 |
| CD4 EM4 | 0.3 (0.2 – 0.4) | 0.5 (0.2 – 1.0) | **0.0262** | 8.7 (7.3 – 11.0) | 12.5 (10.1 – 16.3) | **0.0177** | 7.8 (6.5 – 10.7) | 7.5 (5.2 – 10.4) | 0.8355 |
| CD4 TEMRA | 0.04 (0.02 – 0.08) | 0.03 (0.03 – 0.05) | 0.2678 | 0.14 (0.06 – 0.18) | 0.07 (0.03 – 0.15) | 0.3581 | 0.09 (0.05 – 0.13) | 0.13 (0.04 – 0.18) | 0.4047 |
| CD4 E | None Detected | | | 7.1 (0.0 – 13.3) | 0.0 (0.0 – 16.7) | **0.0478** | 0.0 (0.0 – 0.0) | 0.0 (0.0 – 2.8) | **0.0432** |
| CD4 pE1 | 80.8 (72.7 – 90.0) | 72.5 (60.1 – 82.7) | 0.1188 | 80.0 (75.0 – 93.8) | 71.4 (30.0 – 91.3) | 0.2628 | 33.3 (25.0 – 50.0) | 33.3 (21.6 – 50.0) | 0.6504 |
| CD4 pE2 | 14.8 (7.3 – 25.0) | 17.4 (6.1 – 42.9) | 0.1772 | 0.0 (0.0 – 8.3) | 0.0 (0.0 – 5.6) | 0.7471 | 57.1 (50.0 – 70.8) | 58.8 (42.1 – 76.1) | 0.9248 |

Abbreviations: CD=Cluster of differentiation; EM=Effector memory; HEU=HIV-exposed-uninfected; HUU=HIV-unexposed-uninfected; TEMRA=Terminally differentiated effector memory cells re-expressing CD45RA: E (CD27-CD28-), pE1 (CD27+CD28+), pE2 (CD27+CD28-)

P-Values denoted in bold indicate significance (p<0.05)

Results are presented as median and interquartile range.

**Table S4: Comparison of CD8+ T-cell maturation stages between mothers living with and without HIV at 28 weeks' gestation and at the time of birth**

|  | **Mothers at 28 Weeks’ gestation** | | | **Mothers at the time birth** | | |
| --- | --- | --- | --- | --- | --- | --- |
| **Description** | **MLWH (n=42)** | **MNLWH (n=34)** | **p-Value** | **MLWH (n=33)** | **MNLWH (n=31)** | **p-Value** |
| Total % CD8+ T-cells | 39.9 (26.8 – 52.5) | 23.6 (16.9 – 31.1) | **0.0001** | 45.6 (32.5 – 58.0) | 29.9 (21.9 – 37.3) | **0.0005** |
| CD8 Naive | 27.6 (15.4 – 33.9) | 36.5 (31.2 – 46.1) | **0.0003** | 20.6 (12.4 – 32.9) | 39.5 (22.5 – 52.4) | **0.0049** |
| CD8 Central Memory | 8.1 (6.0 – 9.9) | 7.1 (6.0 – 9.3) | 0.5260 | 7.0 (5.0 – 9.2) | 6.2 (4.8 – 9.8) | 0.9015 |
| CD8 EM | 33.9 (21.9 – 42.1) | 25.9 (19.9 – 30.6) | **0.0157** | 32.5 (19.6 – 40.0) | 22.0 (16.8 – 27.5) | **0.0200** |
| CD8 EM1 | 33.6 (26.9 – 48.2) | 48.9 (37.7 – 64.9) | **0.0066** | 35.8 (24.2 – 52.6) | 38.8 (25.6 – 60.1) | 0.2398 |
| CD8 EM2 | 14.5 (12.0 – 23.6) | 11.3 (7.6 – 14.7) | **0.0045** | 14.6 (10.6 – 22.8) | 14.4 (9.9 – 19.2) | 0.4551 |
| CD8 EM3 | 37.6 (23.9 – 45.8) | 31.2 (17.7 – 37.9) | 0.1918 | 34.6 (24.5 – 50.7) | 35.4 (17.7 – 51.3) | 0.4641 |
| CD8 EM4 | 5.7 (3.3 – 9.8) | 4.6 (3.9 – 8.9) | 0.5321 | 3.7 (2.3 – 8.0) | 3.3 (1.9 – 7.1) | 0.8984 |
| CD8 TEMRA | 28.3 (21.6 – 40.0) | 28.1 (21.7 – 37.7) | 0.9068 | 29.0 (23.8 – 47.9) | 9.8 (25.5 – 40.2) | 0.5864 |
| CD8 E | 76.9 (60.3 – 84.9) | 83.3 (76.9 – 90.1) | **0.0058** | 77.7 (68.3 – 87.3) | 81.5 (73.7 – 85.1) | 0.2017 |
| CD8 pE1 | 3.0 (1.4 – 5.2) | 2.4 (1.7 – 6.5) | 0.4992 | 3.7 (2.1 – 5.1) | 3.9 (2.1 – 5.2) | 0.7488 |
| CD8 pE2 | 17.6 (12.5 – 26.6) | 14.0 (8.2 – 21.1) | **0.0495** | 15.3 (9.6 – 23.9) | 13.4 (8.8 – 18.0) | 0.2307 |

Abbreviations: CD=Cluster of differentiation; EM=Effector memory; MLWH=Mothers living with HIV; MNLWH=Mothers not living with HIV; TEMRA=Terminally differentiated effector memory cells re-expressing CD45RA: E (CD27-CD28-), pE1 (CD27+CD28+), pE2 (CD27+CD28-)

P-Values denoted in bold indicate significance (p<0.05)

Results are presented as median and interquartile range.

**Table S5: Comparison of CD8+ T-cell maturation stages between HIV-exposed and unexposed infants at birth, ten weeks and six months of age**

|  | **Infants at the time of birth** | | | **Infants at ten weeks of age** | | | **Infants at six months age** | | |
| --- | --- | --- | --- | --- | --- | --- | --- | --- | --- |
| **Description** | **HEU (n=28)** | **HUU (n=25)** | **p-Value** | **HEU (n=16)** | **HUU (n=15)** | **p-Value** | **HEU (n=12)** | **HUU (n=15)** | **p-Value** |
| Total % CD8+ T-cells | 21.4 (16.5 – 27.9) | 24.2 (13.3 – 28.7) | 0.7756 | 15.9 (12.5 – 28.5) | 20.3 (10.8 – 28.3) | 0.8433 | 22.3 (18.5 – 25.6) | 29.8 (14.2 – 37.4) | 0.4350 |
| CD8 Naive | 69.4 (57.1 – 77.3) | 63.3 (54.3 – 71.6) | 0.1757 | 41.3 (29.9 – 68.0) | 46.3 (32.4 – 70.8) | 0.6926 | 51.9 (40.3 – 74.3) | 50.6 (38.7 – 65.4) | 0.7482 |
| CD8 Central Memory | 15.1 (10.4 – 19.6) | 20.9 (14.0 – 30.4) | **0.0171** | 7.8 (5.5 – 15.6) | 13.4 (5.1 – 17.1) | 0.4065 | 4.6 (3.1 – 7.1) | 4.6 (2.5 – 7.6) | 0.6179 |
| CD8 EM | 6.0 (4.4 – 10.6) | 8.1 (4.8 – 12.1) | 0.2326 | 24.1 (10.9 – 37.2) | 25.6 (5.4 – 40.5) | 0.8125 | 9.5 (6.3 – 16.3) | 12.8 (8.2 – 20.8) | 0.3346 |
| CD8 EM1 | 49.7 (41.4 – 58.8) | 50.9 (39.8 – 67.4) | 0.4894 | 40.3 (15.3 – 62.2) | 35.1 (18.6 – 58.9) | 0.8744 | 23.5 (13.7 – 34.9) | 18.8 (9.3 – 28.4) | 0.4507 |
| CD8 EM2 | 46.8 (39.0 – 57.7) | 45.7 (28.3 – 55.3) | 0.2732 | 48.0 (35.2 – 67.6) | 54.3 (32.9 – 61.8) | 0.7820 | 48.9 (40.5 – 64.7) | 44.4 (34.7 – 59.8) | 0.2494 |
| CD8 EM3 | 0.4 (0.1 – 0.7) | 0.3 (0.0 – 0.9) | 0.6879 | 1.5 (0.0 – 13.1) | 7.6 (1.3 – 19.5) | 0.2058 | 9.8 (1.3 – 31.1) | 29.6 (13.7 – 35.4) | 0.3262 |
| CD8 EM4 | None Detected | | | 0.0 (0.0 – 0.1) | 0.3 (0.0 – 0.6) | **0.0243** | 0.2 (0.1 – 0.3) | 0.4 (0.0 – 0.8) | 0.2578 |
| CD8 TEMRA | 3.2 (1.9 – 6.5) | 2.8 (1.8 – 4.9) | 0.5206 | 15.4 (4.7 – 20.8) | 11.1 (3.4 – 16.5) | 0.3507 | 14.9 (8.8 – 29.3) | 23.7 (7.2 – 34.8) | 0.3955 |
| CD8 E | 2.0 (0.6 – 3.2) | 2.8 (1.0 – 5.2) | 0.1214 | 7.3 (1.3 – 19.2) | 11.0 (2.1 – 35.8) | 0.2628 | 22.7 (1.9 – 53.5) | 49.8 (9.6 – 61.6) | 0.1567 |
| CD8 pE1 | 29.8 (21.4 – 38.4) | 28.9 (20.2 – 35.2) | 0.5329 | 14.8 (5.0 – 31.7) | 11.5 (3.9 – 19.2) | 0.3132 | 7.8 (3.2 – 25.9) | 4.5 (3.0 – 5.2) | 0.3118 |
| CD8 pE2 | 68.2 (48.7 – 74.5) | 63.3 (58.6 – 72.3) | 0.6169 | 70.4 (64.3 – 76.5) | 62.3 (57.7 – 74.8) | 0.1161 | 62.8 (44.4 – 72.0) | 43.9 (31.9 – 58.6) | 0.1262 |

Abbreviations: CD=Cluster of differentiation; EM=Effector memory; HEU=HIV-exposed-uninfected; HUU=HIV-unexposed-uninfected; TEMRA=Terminally differentiated effector memory cells re-expressing CD45RA: E (CD27-CD28-), pE1 (CD27+CD28+), pE2 (CD27+CD28-)

P-Values denoted in bold indicate significance (p<0.05)

Results are presented as median and interquartile range.

**Table S6: Percentage of PD-1 expression on CD4+ T-cells between mothers living with and without HIV at 28 weeks’ gestation and the time of birth**

|  | **Mothers at 28 Weeks’ gestation** | | | **Mothers at the time birth** | | |
| --- | --- | --- | --- | --- | --- | --- |
| **Description** | **MLWH (n=40)** | **MNLWH (n=31)** | **p-Value** | **MLWH (n=30)** | **MNLWH (n=29)** | **p-Value** |
| Total % CD4+ T-cells | 41.3 (27.2 – 51.6) | 27.9 (22.1 – 32.3) | **0.0005** | 17.5 (12.7 – 24.5) | 16.8 (12.3 – 21.3) | 0.4394 |
| CD4 Naïve | 4.4 (1.8 – 6.8) | 1.8 (1.1 – 3.3) | **0.0006** | 3.9 (2.0 – 8.6) | 2.2 (1.6 – 2.7) | **0.0143** |
| CD4 Central Memory | 63.4 (47.2 – 71.1) | 44.1 (33.6 – 54.5) | **0.0002** | 31.9 (24.7 – 46.3) | 27.2 (21.4 – 34.7) | **0.0499** |
| CD4 EM | 90.2 (80.1 – 93.1) | 77.8 (72.2 – 85.9) | **0.0003** | 72.6 (59.6 – 81.5) | 63.7 (53.2 – 75.7) | 0.1995 |
| CD4 EM1 | 72.3 (62.5 – 77.9) | 62.3 (54.1 – 70.5) | **0.0035** | 61.1 (55.6 – 70.5) | 57.4 (49.1 – 70.9) | 0.2677 |
| CD4 EM2 | None detected | | | 54.6 (25.0 – 77.4) | 41.8 (16.7 – 55.6) | **0.0377** |
| CD4 EM3 | 99.8 (99.6 – 100.0) | 98.8 (97.6 – 99.9) | **0.0075** | 80.9 (50.5 – 94.3) | 80.5 (66.8 – 92.6) | 0.7175 |
| CD4 EM4 | 70.3 (55.5 – 78.5) | 62.1 (50.8 – 72.5) | **0.0395** | 67.4 (52.9 – 82.7) | 64.6 (59.5 – 72.7) | 0.4278 |
| CD4 TEMRA | 71.5 (43.3 – 92.3) | 67.6 (57.1 – 83.3) | 0.9007 | 1.3 (0.7 – 3.3) | 0.9 (0.5 – 1.3) | **0.0313** |
| CD4 E | 84.0 (47.8 – 100) | 75.2 (58.5 – 93.2) | 0.6945 | 0.36 (0.03 – 1.82) | 0.05 (0.00 – 0.10) | **0.0148** |
| CD4 pE1 | 50.0 (30.0 – 100) | 50.0 (25.0 – 66.7) | 0.3841 | 27.9 (13.6 – 48.5) | 20.0 (10.0 – 33.3) | **0.0250** |
| CD4 pE2 | 95.0 (65.5 – 100) | 77.8 (56.8 – 100) | 0.3713 | None detected | | |

Abbreviations: CD=Cluster of differentiation; EM=Effector memory; MLWH=Mothers living with HIV; MNLWH=Mothers not living with HIV; TEMRA=Terminally differentiated effector memory cells re-expressing CD45RA: E (CD27-CD28-), pE1 (CD27+CD28+), pE2 (CD27+CD28-)

P-Values denoted in bold indicate significance (p<0.05)

Results are presented as median and interquartile range.

**Table S7: Percentage of PD-1 expression on CD4+ T-cells between** **HIV-exposed and unexposed infants at birth, ten weeks and six months of age**

|  | **Infants at the time of birth** | | | **Infants at ten weeks of age** | | | **Infants at six months age** | | |
| --- | --- | --- | --- | --- | --- | --- | --- | --- | --- |
| **Description** | **HEU (n=28)** | **HUU (n=25)** | **p-Value** | **HEU (n=16)** | **HUU (n=13)** | **p-Value** | **HEU (n=14)** | **HUU (n=15)** | **p-Value** |
| Total % CD4+ T-cells | 75.2 (68.5 – 81.0) | 73.8 (65.5 – 83.8) | 0.6526 | 20.7 (14.2 – 26.8) | 17.0 (13.0 – 22.0) | 0.3571 | 9.0 (7.4 – 10.4) | 13.6 (11.3 – 24.8) | **0.0019** |
| CD4 Naïve | 48.8 (35.9 – 61.0) | 35.5 (30.4 – 51.1) | 0.0951 | 8.2 (3.0 – 16.0) | 3.6 (2.8 – 7.8) | 0.2274 | 17.8 (12.3 – 22.3) | 23.0 (14.7 – 32.2) | 0.2071 |
| CD4 Central Memory | 44.9 (34.1 – 62.3) | 59.6 (35.9 – 63.5) | 0.2882 | 16.8 (8.6 – 26.7) | 12.9 (7.0 – 19.7) | 0.8433 | 18.8 (14.4 – 23.3) | 23.0 (11.1 – 36.4) | 0.4168 |
| CD4 EM | 2.8 (2.1 – 3.5) | 2.3 (1.5 – 3.8) | 0.3848 | 46.1 (39.9 – 56.1) | 51.0 (38.8 – 66.0 | 0.3633 | 52.2 (42.8 – 57.2) | 59.7 (54.8 – 72.1) | **0.0102** |
| CD4 EM1 | 96.4 (95.1 – 97.3) | 94.3 (87.5 – 97.1) | **0.0376** | 44.1 (38.8 – 50.5) | 49.8 (39.5 – 61.4) | 0.2517 | 44.8 (37.0 – 52.4) | 52.3 (45.0 – 61.8) | **0.0434** |
| CD4 EM2 | 2.6 (2.0 – 4.1) | 4.0 (2.1 – 8.1) | 0.1309 | 27.5 (16.7 – 66.7) | 45.5 (14.3 – 71.0) | 0.7369 | 33.1 (26.8 – 43.8) | 39.3 (34.3 – 50.9) | **0.0259** |
| CD4 EM3 | None detected | | | 76.9 (49.0 – 100.0) | 83.9 (68.6 – 92.5) | 0.2917 | 33.3 (22.2 – 57.1) | 43.3 (25.0 – 50.0) | 0.4273 |
| CD4 EM4 | 0.3 (0.2 – 0.4) | 0.5 (0.2 – 1.0) | 0.0525 | 41.7 (32.3 – 49.3) | 40.0 (32.6 – 67.7) | 0.4768 | 40.7 (34.2 – 44.7) | 54.4 (38.5 – 64.9) | **0.0356** |
| CD4 TEMRA | 0.04 (0.02 – 0.08) | 0.03 (0.03 – 0.05) | 0.2678 | 27.9 (9.4 – 36.8) | 16.7 (0.0 – 50.0) | 0.5127 | 12.5 (8.3 – 26.7) | 11.8 (2.6 – 22.2) | 0.8502 |
| CD4 E | None detected | | | 0.0 (0.0 – 50.0) | 2.6 (0.0 – 75.0) | 0.2976 | 50.0 (0.0 – 100.0) | 75.0 (0.0 – 100.0) | 0.9518 |
| CD4 pE1 | 80.8 (72.7 – 90.0) | 72.5 (60.1 – 82.7) | 0.1188 | 40.3 (33.3 – 56.1) | 33.3 (8.3 – 33.3) | **0.0457** | 33.3 (12.5 – 50.0) | 34.6 (16.7 – 66.7) | 0.4388 |
| CD4 pE2 | 14.8 (7.3 – 25.0) | 17.4 (6.1 – 42.9) | 0.1772 | 40.0 (0.0 – 100.0) | 20.0 (0.0 – 100.0) | 0.7983 | None detected | | |

Abbreviations: CD=Cluster of differentiation; EM=Effector memory; HEU=HIV-exposed-uninfected; HUU=HIV-unexposed-uninfected; TEMRA=Terminally differentiated effector memory cells re-expressing CD45RA: E (CD27-CD28-), pE1 (CD27+CD28+), pE2 (CD27+CD28-)

P-Values denoted in bold indicate significance (p<0.05)

Results are presented as median and interquartile range.

**Table S8: Percentage of CD57 expression on CD4+ T-cells between mothers living with and without HIV at 28 weeks’ gestation**

|  | **Mothers at 28 Weeks’ gestation** | | | **Mothers at the time birth** | | |
| --- | --- | --- | --- | --- | --- | --- |
| **Description** | **MLWH (n=34)** | **MNLWH (n=32)** | **p-Value** | **MLWH (n=31)** | **MNLWH (n=27)** | **p-Value** |
| Total % CD4+ T-cells | 4.8 (2.6 – 6.7) | 2.7 (1.4 – 5.6) | **0.0271** | 14.1 (8.1 – 26.5) | 9.9 (7.7 – 12.0) | **0.0124** |
| CD4 Naïve | None detected | | | 0.08 (0.06 – 0.17) | 0.03 (0.01 – 0.09) | **0.0141** |
| CD4 Central Memory | 0.4 (0.3 – 0.6) | 0.3 (0.2 – 0.4) | **0.0376** | 0.3 (0.2 – 0.4) | 0.3 (0.1 – 0.3) | 0.2366 |
| CD4 EM | 22.4 (16.5 – 38.3) | 15.7 (6.7 – 26.7) | **0.0058** | 40.4 (21.3 – 52.9) | 24.2 (13.0 – 41.4) | **0.0181** |
| CD4 EM1 | 2.2 (1.7 – 3.4) | 2.3 (1.1 – 3.0) | 0.6127 | 2.5 (1.0 – 4.6) | 2.3 (1.3 – 4.7) | 0.6779 |
| CD4 EM2 | 45.0 (25.0 – 70.0) | 43.5 (22.2 – 66.7) | 0.5832 | 67.6 (26.7 – 79.3) | 49.4 (16.7 – 66.7) | **0.0256** |
| CD4 EM3 | 75.1 (62.6 – 83.2) | 71.3 (57.7 – 79.7) | 0.3472 | 70.8 (53.4 – 83.8) | 69.9 (54.7 – 79.4) | 0.7170 |
| CD4 EM4 | 6.5 (3.3 – 12.0) | 4.8 (2.7 – 7.5) | **0.0397** | 16.4 (7.2 – 27.9) | 10.7 (7.5 – 25.4) | 0.4832 |
| CD4 TEMRA | 43.9 (13.4 – 73.3) | 37.7 (18.0 – 66.4) | 0.6102 | 49.6 (42.0 – 55.6) | 46.4 (37.2 – 54.0) | 0.2204 |
| CD4 E | 58.7 (26.7 – 86.9) | 59.1 (35.0 – 80.0) | 0.7281 | 50.8 (44.5 – 59.0) | 49.2 (38.4 – 58.5) | 0.3038 |
| CD4 pE1 | 0.0 (0.0 – 25.0) | 0.0 (0.0 – 0.0) | **0.0070** | None detected | | |
| CD4 pE2 | 0.0 (0.0 – 26.8) | 5.7 (0.0 – 33.3) | 0.4282 |  |  |  |

Abbreviations: CD=Cluster of differentiation; EM=Effector memory; MLWH=Mothers living with HIV; MNLWH=Mothers not living with HIV; TEMRA=Terminally differentiated effector memory cells re-expressing CD45RA: E (CD27-CD28-), pE1 (CD27+CD28+), pE2 (CD27+CD28-)

P-Values denoted in bold indicate significance (p<0.05)

Results are presented as median and interquartile range.

**Table S9: Percentage of CD57 expression on CD4+ T-cells HIV-exposed and unexposed infants at birth, ten weeks and six months of age**

|  | **Infants at the time of birth** | | | **Infants at ten weeks of age** | | | **Infants at six months age** | | |
| --- | --- | --- | --- | --- | --- | --- | --- | --- | --- |
| **Description** | **HEU (n=23)** | **HUU (n=24)** | **p-Value** | **HEU (n=14)** | **HUU (n=13)** | **p-Value** | **HEU (n=15)** | **HUU (n=14)** | **p-Value** |
| Total % CD4+ T-cells | 0.05 (0.03 – 0.07) | 0.03 (0.02 – 0.06) | **0.0495** | 0.2 (0.1 – 0.4) | 0.3 (0.1 – 0.4) | 0.9227 | 0.02 (0.01 – 0.06) | 0.07 (0.04 – 0.09) | **0.0026** |
| CD4 Naïve | 0.16 (0.08 – 0.25) | 0.10 (0.04 – 0.16) | **0.0397** | 0.5 (0.2 – 1.7) | 0.3 (0.2 – 0.5) | 0.2790 | 12.8 (10.4 – 18.0) | 20.5 (13.6 – 27.1) | **0.0294** |
| CD4 Central Memory | 4.7 (1.1 – 7.5) | 2.4 (0.9 – 6.6) | 0.3068 | 0.3 (0.1 – 0.7) | 0.3 (0.1 – 0.4) | 0.5127 | 13.2 (9.8 – 16.5) | 19.6 (12.8 – 26.5) | **0.0184** |
| CD4 EM | 1.5 (0.8 – 1.8) | 1.1 (0.5 – 1.9) | 0.6785 | 0.7 (0.2 – 2.9) | 0.9 (0.4 – 3.0) | 0.5351 | 0.5 (0.3 – 1.3) | 1.1 (0.5 – 1.7) | 0.1051 |
| CD4 EM1 | 1.4 (0.8 – 1.8) | 1.1 (0.5 – 1.6) | 0.3180 | 0.9 (0.5 – 1.4) | 1.0 (0.4 – 1.8) | 0.8195 | 0.4 (0.3 – 1.0) | 1.1 (0.5 – 1.4) | 0.0883 |
| CD4 EM2 | None detected | | | 0.0 (0.0 – 14.3) | 0.0 (0.0. – 0.0) | **0.0236** | 0.7 (0.0 – 1.9) | 0.7 (0.3 – 1.3) | 0.7811 |
| CD4 EM3 |  |  |  | 20.8 (0.0 – 41.2) | 22.0 (6.4 – 33.3) | 0.8918 | 0.0 (0.0 – 0.0) | 0.0 (0.0 – 1.6) | 0.4602 |
| CD4 EM4 |  |  |  | 1.1 (0.0 – 3.7) | 1.1 (0.0 – 2.8) | 0.7369 | None detected | | |
| CD4 TEMRA |  |  |  | None detected | | | 0.0 (0.0 – 5.0) | 2.8 (0.0 – 6.1) | 0.5647 |
| CD4 E |  |  |  |  |  |  | None detected | | |
| CD4 pE1 |  |  |  |  |  |  | 0.0 (0.0 – 0.0) | 0.0 (0.0 – 7.7) | **0.0416** |
| CD4 pE2 |  |  |  |  |  |  | None detected | | |

Abbreviations: CD=Cluster of differentiation; EM=Effector memory; HEU=HIV-exposed-uninfected; HUU=HIV-unexposed-uninfected; TEMRA=Terminally differentiated effector memory cells re-expressing CD45RA: E (CD27-CD28-), pE1 (CD27+CD28+), pE2 (CD27+CD28-)

P-Values denoted in bold indicate significance (p<0.05)

Results are presented as median and interquartile range.

**Table S10: Percentage of PD-1 expression on CD8+ T-cells between mothers living with and without HIV at 28 weeks’ gestation and the time of birth**

|  | **Mothers at 28 Weeks’ gestation** | | | **Mothers at the time birth** | | |
| --- | --- | --- | --- | --- | --- | --- |
| **Description** | **MLWH (n=42)** | **MNLWH (n=33)** | **p-Value** | **MLWH (n=32)** | **MNLWH (n=30)** | **p-Value** |
| Total % CD8+ T-cells | 51.8 (39.1 – 62.3) | 33.9 (26.5 – 44.4) | **0.0001** | 22.5 (11.5 – 32.0) | 13.6 (10.5 – 19.9) | **0.0117** |
| CD8 Naive | 9.6 (6.7 – 14.6) | 4.8 (3.4 – 8.1) | **0.0002** | 4.1 (2.7 – 10.4) | 1.3 (0.9 – 3.0) | **0.0001** |
| CD8 Central Memory | 62.4 (53.2 – 71.4) | 51.8 (43.9 – 58.6) | **0.0001** | 39.1 (32.0 – 49.9) | 30.5 (24.8 – 41.6) | **0.0249** |
| CD8 EM | 64.1 (52.7 – 74.3) | 60.1 (43.8 – 72.1) | 0.1786 | 42.8 (27.4 – 51.7) | 34.7 (28.3 – 43.1) | 0.1468 |
| CD8 EM1 | 62.9 (50.2 – 74.6) | 41.9 (29.1 – 64.3) | **0.0013** | 53.1 (44.4 – 62.0) | 45.4 (35.2 – 52.4) | **0.0420** |
| CD8 EM2 | 47.7 (35.4 – 58.0) | 36.8 (30.9 – 51.3) | **0.0321** | 76.1 (67.8 – 82.4) | 60.5 (50.0 – 75.9) | **0.0076** |
| CD8 EM3 | 20.3 (12.3 – 26.0) | 16.3 (9.5 – 32.4) | 0.6139 | 51.1 (29.1 – 64.2) | 38.3 (26.3 – 52.3) | 0.1318 |
| CD8 EM4 | 20.3 (10.7 – 29.6) | 10.1 (0.0 – 25.0) | **0.0291** | 35.4 (26.7 – 53.7) | 42.1 (32.0 – 57.9) | 0.1934 |
| CD8 TEMRA | 33.9 (28.7 – 49.6) | 27.5 (21.6 – 42.1) | **0.0256** | 17.0 (7.9 – 24.2) | 13.0 (8.5 – 17.1) | 0.1188 |
| CD8 E | 29.4 (23.3 – 38.9) | 24.4 (19.2 – 39.6) | 0.3246 | 8.6 (3.3 – 15.7) | 6.9 (3.7 – 10.8) | 0.2662 |
| CD8 pE1 | 3.3 (2.0 – 5.0) | 2.3 (1.3 – 3.6) | **0.0440** | 40.4 (29.7 – 53.3) | 41.9 (33.3 – 59.4) | 0.2480 |
| CD8 pE2 | 7.0 (5.2 – 8.0) | 5.2 (3.1 – 7.3) | **0.0117** | 29.9 (17.8 – 40.0) | 29.8 (18.0 – 38.3) | 0.9886 |

Abbreviations: CD=Cluster of differentiation; EM=Effector memory; MLWH=Mothers living with HIV; MNLWH=Mothers not living with HIV; TEMRA=Terminally differentiated effector memory cells re-expressing CD45RA: E (CD27-CD28-), pE1 (CD27+CD28+), pE2 (CD27+CD28-)

P-Values denoted in bold indicate significance (p<0.05)

Results are presented as median and interquartile range.

**Table S11: Percentage of PD-1 expression on CD8+ T-cells between** **HIV-exposed and unexposed infants at birth, ten weeks and six months of age**

|  | **Infants at the time of birth** | | | **Infants at ten weeks of age** | | | **Infants at six months age** | | |
| --- | --- | --- | --- | --- | --- | --- | --- | --- | --- |
| **Description** | **HEU (n=29)** | **HUU (n=26)** | **p-Value** | **HEU (n=16)** | **HUU (n=15)** | **p-Value** | **HEU (n=17)** | **HUU (n=15)** | **p-Value** |
| Total % CD8+ T-cells | 33.3 (21.3 – 49.6) | 34.7 (18.6 – 43.8) | 0.9328 | 39.0 (22.6 – 47.7) | 36.2 (22.3 – 52.6) | 0.8744 | 14.7 (5.1 – 22.3) | 13.4 (6.1 – 21.6) | 0.9220 |
| CD8 Naive | 21.3 (15.1 – 41.4) | 26.3 (15.6 – 32.7) | 0.9862 | 20.2 (8.1 – 32.8) | 13.4 (11.3 – 32.7) | 0.7270 | 13.4 (11.5 – 18.0) | 16.9 (10.7 – 21.0) | 0.3695 |
| CD8 Central Memory | 35.8 (22.9 – 51.0) | 31.7 (16.4 – 41.5) | 0.3899 | 14.0 (8.4 – 22.8) | 9.2 (6.7 – 12.8) | 0.3604 | 12.4 (8.2 – 16.3) | 8.2 (6.7 – 20.5) | 0.3356 |
| CD8 EM | 58.0 (50.4 – 65.1) | 57.8 (49.6 – 61.0) | 0.8977 | 33.6 (23.6 – 40.7) | 35.7 (31.4 – 62.6) | 0.2357 | 28.6 (23.2 – 38.7) | 25.4 (19.2 – 41.2) | 0.8433 |
| CD8 EM1 | 55.9 (47.6 – 63.2) | 58.0 (49.7 – 65.8) | 0.6210 | 29.6 (23.9 – 47.9) | 32.3 (28.3 – 52.4) | 0.4526 | 42.4 (33.9 – 62.0) | 40.7 (30.2 – 56.9) | 0.3549 |
| CD8 EM2 | 47.1 (35.4 – 58.4) | 43.0 (40.8 – 50.8) | 0.4668 | 35.1 (28.2 – 41.4) | 41.9 (33.7 – 56.4) | 0.1331 | 27.3 (20.6 – 40.6) | 29.7 (23.2 – 43.7) | 0.5840 |
| CD8 EM3 | 92.3 (49.2 – 100.0) | 83.3 (41.7 – 100.0) | 0.4782 | 20.0 (18.2 – 31.3) | 20.8 (35.4 – 49.3) | **0.0313** | 31.5 (11.8 – 50.9) | 26.1 (19.3 – 36.9) | 0.6592 |
| CD8 EM4 | None detected | | | 33.3 (20.0 – 50.0) | 11.1 (0.0 – 50.0) | 0.2050 | 12.0 (0.0 – 50.0) | 33.3 (21.4 – 38.5) | 0.5711 |
| CD8 TEMRA | 54.0 (49.5 – 60.7) | 52.6 (44.8 – 59.4) | 0.5212 | 36.4 (26.1 – 48.8) | 41.5 (29.7 – 48.9) | 0.4768 | 25.2 (15.8 – 32.6) | 30.0 (20.4 – 40.9) | 0.2990 |
| CD8 E | 37.5 (7.9 – 50.0) | 33.3 (11.1 – 50.0) | 0.8648 | 28.2 (16.3 – 47.4) | 31.9 (18.8 – 45.0) | 0.6997 | 1.6 (0.0 – 3.8) | 10.9 (3.7 – 16.1) | **0.0066** |
| CD8 pE1 | 51.5 (39.6 – 59.6) | 60.1 (40.8 – 72.4) | 0.3112 | 41.8 (22.1 – 55.7) | 45.8 (32.7 – 54.6) | 0.5936 | 44.6 (34.2 – 53.6) | 44.4 (28.0 – 49.2) | 0.5800 |
| CD8 pE2 | 56.5 (43.2 – 65.3) | 51.0 (41.7 – 60.8) | 0.4024 | 36.8 (26.2 – 44.2) | 45.6 (30.2 – 52.2) | 0.2059 | 19.4 (13.7 – 27.3) | 24.3 (17.7 – 39.0) | 0.1460 |

Abbreviations: CD=Cluster of differentiation; EM=Effector memory; HEU=HIV-exposed-uninfected; HUU=HIV-unexposed-uninfected; TEMRA=Terminally differentiated effector memory cells re-expressing CD45RA: E (CD27-CD28-), pE1 (CD27+CD28+), pE2 (CD27+CD28-)

P-Values denoted in bold indicate significance (p<0.05)

Results are presented as median and interquartile range.

**Table S12: Percentage of CD57 expression on CD8+ T-cells between mothers living with and without HIV at 28 weeks’ gestation**

|  | **Mothers at 28 Weeks’ gestation** | | | **Mothers at the time birth** | | |
| --- | --- | --- | --- | --- | --- | --- |
| **Description** | **MLWH (n=41)** | **MNLWH (n=34)** | **p-Value** | **MLWH (n=33)** | **MNLWH (n=31)** | **p-Value** |
| Total % CD8+ T-cells | 27.7 (18.3 – 33.6) | 24.5 (16.3 – 31.3) | 0.2016 | 28.2 (17.5 – 37.4) | 21.1 (13.8 – 27.2) | **0.0367** |
| CD8 Naive | 7.5 (5.5 – 10.8) | 3.6 (2.5 – 6.6) | **0.0001** | 0.4 (0.3 – 0.7) | 0.3 (0.2 – 0.4) | **0.0266** |
| CD8 Central Memory | 2.9 (1.9 – 3.9) | 0.9 (0.5 – 2.0) | **0.0001** | 0.8 (0.6 – 1.8) | 0.2 (0.0 – 0.5) | **0.0001** |
| CD8 EM | 34.3 (22.9 – 40.3) | 26.7 (17.6 – 40.5) | 0.3305 | 38.1 (24.2 – 49.8) | 40.1 (23.4 – 51.9) | 0.6527 |
| CD8 EM1 | 5.9 (3.4 – 9.3) | 3.4 (2.3 – 4.9) | **0.0010** | None detected | | |
| CD8 EM2 | 31.2 (22.9 – 37.2) | 34.8 (25.4 – 46.9) | 0.1072 | 41.9 (34.0 – 57.4) | 54.3 (43.7 – 67.1) | **0.0171** |
| CD8 EM3 | 65.3 (54.4 – 77.6) | 71.9 (55.2 – 81.3) | 0.2927 | 79.1 (67.3 – 85.3) | 76.7 (69.4 – 82.9) | 0.6040 |
| CD8 EM4 | 8.0 (3.3 – 15.1) | 0.0 (0.0 – 11.8) | **0.0326** | 16.7 (11.5 – 26.7) | 12.8 (4.0 – 22.2) | **0.0325** |
| CD8 TEMRA | 52.0 (44.4 – 63.3) | 56.0 (46.8 – 66.5) | 0.2414 | 42.6 (33.7 – 51.8) | 38.3 (32.2 – 47.9) | 0.2591 |
| CD8 E | 46.5 (38.0 – 60.2) | 48.7 (41.7 – 60.7) | 0.2548 | 51.7 (37.6 – 64.0) | 39.5 (30.4 – 53.9) | **0.0142** |
| CD8 pE1 | 0.2 (0.2 – 0.3) | 0.2 (0.1 – 0.4) | 0.7076 | None detected | | |
| CD8 pE2 | 3.3 (2.0 – 6.3) | 3.1 (2.1 – 4.6) | 0.4630 | 29.0 (17.8 – 41.5) | 27.9 (19.1 – 41.9) | 0.9198 |

Abbreviations: CD=Cluster of differentiation; EM=Effector memory; MLWH=Mothers living with HIV; MNLWH=Mothers not living with HIV; TEMRA=Terminally differentiated effector memory cells re-expressing CD45RA: E (CD27-CD28-), pE1 (CD27+CD28+), pE2 (CD27+CD28-)

P-Values denoted in bold indicate significance (p<0.05)

Results are presented as median and interquartile range.

**Table S13: Percentage of CD57 expression on CD8+ T-cells between HIV-exposed and unexposed infants at birth, ten weeks and six months of age**

|  | **Infants at the time of birth** | | | **Infants at ten weeks of age** | | | **Infants at six months age** | | |
| --- | --- | --- | --- | --- | --- | --- | --- | --- | --- |
| **Description** | **HEU (n=20)** | **HUU (n=17)** | **p-Value** | **HEU (n=14)** | **HUU (n=14)** | **p-Value** | **HEU (n=16)** | **HUU (n=15)** | **p-Value** |
| Total % CD8+ T-cells | 0.04 (0.03 – 0.06) | 0.03 (0.01 – 0.04) | **0.0046** | 1.8 (0.2 – 7.4) | 2.8 (0.2 – 4.8) | 0.4907 | 3.9 (0.1 – 14.3) | 12.0 (0.8 – 15.8) | 0.4065 |
| CD8 Naive | 3.0 (0.8 – 7.6) | 2.2 (1.0 – 9.8) | 0.9328 | 23.3 (13.0 – 30.4) | 22.2 (13.8 – 36.4) | 0.6632 | 11.9 (6.9 – 15.7) | 19.2 (11.0 – 29.1) | **0.0402** |
| CD8 Central Memory | 0.9 (0.2 – 1.7) | 0.4 (0.2 – 0.6) | **0.0442** | None detected | | | 3.9 (1.4 – 6.9) | 5.6 (4.2 – 6.7) | 0.2052 |
| CD8 EM | 0.0 (0.0 – 0.1) | 0.0 (0.0 – 0.0) | **0.0027** | 2.4 (0.0 – 7.0) | 2.4 (1.3 – 9.8) | 0.6312 | 4.8 (0.6 – 16.3) | 13.8 (2.7 – 18.7) | 0.2059 |
| CD8 EM1 | 0.0 (0.0 – 0.3) | 0.0 (0.0 – 0.0) | **0.0184** | 0.0 (0.0 – 0.6) | 0.0 (0.0 – 0.0) | **0.0250** | 0.0 (0.0 – 0.6) | 0.2 (0.0 – 0.8) | 0.4450 |
| CD8 EM2 | None detected | | | 6.4 (0.0 – 13.7) | 4.7 (0.0 – 5.4) | 0.3370 | 3.2 (0.3 – 15.1) | 14.0 (2.4 – 23.4) | **0.0495** |
| CD8 EM3 |  |  |  | 14.2 (12.5 – 20.0) | 6.1 (1.5 – 20.6) | 0.1135 | 25.0 (3.7 – 47.4) | 30.9 (20.0 – 36.2) | 0.6157 |
| CD8 EM4 |  |  |  | None detected | | | 8.3 (0.0 – 33.3) | 0.0 (0.0 – 23.1) | 0.7359 |
| CD8 TEMRA |  |  |  | 9.6 (3.1 – 17.9) | 5.6 (2.1 – 13.7) | 0.3714 | 17.2 (1.6 – 27.3) | 22.7 (7.3 – 32.9) | 0.7482 |
| CD8 E | 0.0 (0.0 – 25.0) | 0.0 (0.0 – 0.0) | **0.0040** | 16.2 (4.1 – 26.1) | 14.3 (4.8 – 32.1) | 0.9795 | 19.0 (0.0 – 28.5) | 26.2 (10.2 – 34.9) | 0.4065 |
| CD8 pE1 | None detected | | | None detected | | | None detected | | |
| CD8 pE2 |  |  |  | 8.4 (1.2 – 13.8) | 5.8 (1.1 – 7.5) | **0.0457** | 17.0 (2.0 – 29.8) | 20.8 (7.7 – 32.0) | 0.7482 |

Abbreviations: CD=Cluster of differentiation; EM=Effector memory; HEU=HIV-exposed-uninfected; HUU=HIV-unexposed-uninfected; TEMRA=Terminally differentiated effector memory cells re-expressing CD45RA: E (CD27-CD28-), pE1 (CD27+CD28+), pE2 (CD27+CD28-)

P-Values denoted in bold indicate significance (p<0.05)

Results are presented as median and interquartile range.

**Table S14: Percentage of regulatory T-cell marker expression on CD4+T-cells between mothers living with and without HIV at the time of birth**

|  | **Mothers at 28 Weeks’ gestation** | | | **Mothers at the time birth** | | |
| --- | --- | --- | --- | --- | --- | --- |
| **Description** | **MLWH (n=28)** | **MNLWH (n=22)** | **p-Value** | 39.7 (12.9)* | 57.3 (6.9)* | **0.0001** |
| Total % CD4+ T-cells | 44.4 (33.8 – 51.1) | 57.3 (49.4 – 60.7) | **0.0001** | 16.3 (13.2 – 23.7) | 25.9 (14.6 – 29.7) | **0.0425** |
| CD25 | 25.4 (17.2 – 35.7) | 20.1 (16.1 – 22.6) | **0.0374** | 2.6 (2.5 – 2.7) | 1.9 (1.3 – 2.8) | 0.1514 |
| FoxP3 | 4.4 (3.2 – 5.7) | 3.7 (2.3 – 4.8) | **0.0449** | 3.8 (2.5 – 6.5) | 4.5 (3.9 – 7.7) | 0.3393 |
| CD25+FoxP3+ (Tregs) | 4.3 (2.7 – 7.4) | 2.5 (1.8 – 4.6) | **0.0084** | 4.3 (1.8 – 7.9) | 3.1 (2.3 – 5.2) | 0.4229 |
| CD39 | 4.6 (2.1 – 7.1) | 3.0 (1.5 – 5.1) | 0.1237 | 36.0 (21.3 – 52.6) | 21.9 (16.0 – 25.1) | **0.0134** |
| CD39+ Tregs | 32.5 (14.3 – 59.4) | 24.1 (13.3 – 36.8) | 0.0786 | 4.7 (3.6 – 7.3) | 3.4 (2.9 – 4.9) | **0.0301** |
| Helios | 6.3 (1.4)* | 5.0 (1.5)* | **0.0102** | 0.3 (0.2 – 0.7) | 0.2 (0.1 – 0.3) | **0.0335** |
| FoxP3+Helios+ | 0.6 (0.3 – 0.8) | 0.5 (0.3 – 0.7) | 0.7250 | 70.9 (49.4 – 82.5) | 58.0 (42.4 – 69.3) | 0.1148 |
| Helios+ Tregs | 72.6 (16.1)* | 76.5 (13.0)* | 0.6056 | 36.9 (19.9 – 49.5) | 37.0 (22.8 – 69.6) | 0.7491 |
| CD45RA | 62.7 (26.1 – 97.8) | 27.7 (17.9 – 53.9) | **0.0105** | 0.0 (0.0 – 0.0) | 0.0 (0.0 – 0.2) | 0.1547 |
| CD45RA+ Tregs | 0.8 (0.1 – 4.0) | 0.7 (0.0 – 1.4) | 0.2416 | 39.7 (12.9)* | 57.3 (6.9)* | **0.0001** |

Abbreviations: CD=Cluster of differentiation; FoxP3=Forkhead box protein P3; MLWH=Mothers living with HIV; MNLWH=Mothers not living with HIV

P-Values denoted in bold indicate significance (p<0.05)

Results are presented as median and interquartile range, except when indicated by * in which case results are presented as mean and standard deviation.

**Table S15: Percentage of regulatory T-cell marker expression on CD4+T-cells between** **HIV-exposed and unexposed infants from birth to six months of age**

|  | **Infants at the time of birth** | | | **Infants at ten weeks of age** | | | **Infants at six months age** | | |
| --- | --- | --- | --- | --- | --- | --- | --- | --- | --- |
| **Description** | **HEU (n=24)** | **HUU (n=21)** | **p-Value** | **HEU (n=15)** | **HUU (n=20)** | **p-Value** | **HEU (n=17)** | **HUU (n=15)** | **p-Value** |
| Total % CD4+ T-cells | 70.3 (67.5 – 74.8) | 71.6 (68.3 – 78.5) | 0.6655 | 63.4 (57.1 – 73.9) | 68.3 (60.6 – 74.7) | 0.5709 | 68.3 (61.6 – 72.1) | 61.6 (53.0 – 72.1) | 0.2494 |
| CD25 | 13.5 (12.2 – 14.5) | 11.8 (10.0 – 20.4) | 0.3148 | 10.8 (2.0)* | 11.2 (2.7)* | 0.6623 | 11.9 (10.4 – 15.3) | 11.8 (9.1 – 16.5) | 0.9683 |
| FoxP3 | 2.2 (1.3 – 4.2) | 13.9 (4.8 – 85.0) | **0.0013** | 2.7 (0.7 – 3.9) | 2.9 (1.3 – 4.6) | 0.2861 | 1.1 (1.0 – 1.4) | 1.5 (1.2 – 3.0) | **0.0380** |
| CD25+FoxP3+ (Tregs) | 5.7 (3.2 – 8.3) | 5.7 (2.9 – 8.8) | 0.9999 | 5.3 (1.9)* | 5.9 (2.1)* | 0.6358 | 5.2 (4.4 – 6.0) | 5.4 (4.5 – 6.0) | 0.6979 |
| CD39 | 2.3 (1.4 – 3.1) | 2.1 (1.3 – 2.9) | 0.5024 | 2.2 (1.0)* | 1.9 (1.0)* | 0.3551 | 1.9 (0.5)* | 2.1 (1.5)* | 0.5430 |
| CD39+ Tregs | 18.8 (14.6 – 27.5) | 24.1 (13.1 – 36.6) | 0.2674 | 14.6 (7.1)* | 29.2)* | 0.2124 | 17.8 (9.6)* | 15.1 (11.8)* | 0.4472 |
| Helios | 3.6 (2.0 – 6.3) | 2.9 (1.2 – 4.8) | 0.1279 | 7.6 (2.6)* | 7.8 (4.7)* | 0.5439 | 5.3 (2.7)* | 6.8 (2.4)* | 0.1171 |
| FoxP3+Helios+ | 0.2 (0.1 – 0.3) | 0.3 (0.1 – 0.9) | **0.0485** | 0.3 (0.2 – 0.6) | 0.4 (0.2 – 0.6) | 0.6917 | 0.3 (0.2)* | 0.4 (0.2)* | 0.1200 |
| Helios+ Tregs | 46.3 (29.1 – 69.7) | 50.0 (27.4 – 69.1) | 0.8222 | 64.7 (12.6)* | 64.2 (22.1)* | 0.8031 | 53.6 (26.0)* | 64.3 (14.9)* | 0.2729 |
| CD45RA | 2.3 (1.2 – 4.9) | 1.8 (1.0 – 3.8) | 0.4658 | 25.4 (11.8 – 54.8) | 60.7 (23.9 – 91.0) | 0.1188 | 56.8 (31.0 – 95.5) | 55.0 (34.7 – 94.4) | 0.9423 |
| CD45RA+ Tregs | 0.00 (0.00 – 0.00) | 0.00 (0.00 – 0.08) | 0.0956 | 0.21 (0.11 – 0.27) | 1.0 (0.3 – 1.8) | **0.0209** | 0.08 (0.00 – 0.73) | 0.11 (0.00 – 0.28) | 0.6592 |

Abbreviations: CD=Cluster of differentiation; FoxP3=Forkhead box protein P3; HEU=HIV-exposed-uninfected; HUU=HIV-unexposed-uninfected

P-Values denoted in bold indicate significance (p<0.05)

Results are presented as median and interquartile range, except when indicated by * in which case results are presented as mean and standard deviation.

**Table S16: Percentage of monocyte marker expression between mothers living with and without HIV at 28 weeks’ gestation and at the time of birth**

| **28 Weeks’ gestation** | | | | | | | | | |
| --- | --- | --- | --- | --- | --- | --- | --- | --- | --- |
|  | **Classical** | | | **Intermediate** | | | **Non-classical** | | |
| **Description** | **MLWH (n=37)** | **MNLWH (n=26)** | **p-Value** | **MLWH (n=40)** | **MNLWH (n=28)** | **p-Value** | **MLWH (n=37)** | **MNLWH (n=32)** | **p-Value** |
| Total % CD14+ CL | 79.4 (73.4 – 81.1) | 78.0 (73.2 – 80.8) | 0.5958 | 3.0 (1.9 – 5.7) | 1.8 (0.9 – 3.9) | **0.0151** | 15.4 (4.8)* | 16.0 (7.0)* | 0.5965 |
| CCR2 | 75.8 (70.1 – 79.8) | 73.3 (71.2 – 78.3) | 0.4279 | 3.0 (1.9 – 5.7) | 1.8 (0.9 – 3.9) | **0.0151** | 0.5 (0.3 – 0.8) | 0.5 (0.2 – 1.9) | 0.2411 |
| CD80 | 0.02 (0.01 – 0.04) | 0.01 (0.00 – 0.03) | 0.0770 | 0.4 (0.2 – 0.7) | 0.10 (0.09 – 0.20) | **0.0003** | 0.5 (0.1 – 0.9) | 0.3 (0.1 – 0.5) | 0.1635 |
| CD86 | 62.2 (13.4)* | 58.8 (17.4)* | 0.3059 | 3.0 (1.9 – 5.7) | 1.8 (0.9 – 3.9) | **0.0151** | 14.6 (12.2 – 20.5) | 15.5 (11.5 – 21.1) | 0.8763 |
| TLR3 | 75.7 (6.2)* | 75.1 (5.0)* | 0.4679 | 3.0 (1.9 – 5.7) | 1.8 (0.9 – 3.9) | **0.0151** | 1.7 (0.9 – 3.3) | 2.3 (0.6 – 4.5) | 0.8828 |
| TLR4 | 0.2 (0.1 – 0.5) | 0.2 (0.1 – 0.6) | 0.6879 | 0.09 (0.01 – 0.36) | 0.03 (0.01 – 0.15) | 0.1471 | 0.9 (0.4 – 1.5) | 1.7 (0.4 – 2.7) | 0.1808 |
| PD-L1 | 0.8 (0.5 – 1.6) | 0.9 (0.5 – 1.4) | 0.9517 | 0.3 (0.1 – 0.4) | 0.4 (0.1 – 0.8) | 0.3169 | 0.11 (0,06 – 0.15) | 0.11 (0.06 – 0.15) | 0.6015 |
| PD-L2 | 0.2 (0.1 – 0.3) | 0.2 (0.1 – 0.3) | 0.8503 | 0.5 (0.3 – 1.0) | 0.5 (0.2 – 0.6) | 0.2083 | 0.4 (0.2 – 0.9) | 0.8 (0.2 – 2.1) | 0.1083 |
| **Time of Birth** | | | | | | | | | |
|  | **Classical** | | | **Intermediate** | | | **Non-classical** | | |
| **Description** | **MLWH (n=32)** | **MNLWH (n=30)** | **p-Value** | **MLWH (n=32)** | **MNLWH (n=29)** | **p-Value** | **MLWH (n=32)** | **MNLWH (n=30)** | **p-Value** |
| Total % CD14+ CL | 70.7 (8.4)* | 71.7 (13.1)* | 0.4220 | 11.5 (5.6 – 17.6) | 5.8 (2.4 – 12.4) | 0.1369 | 13.1 (8.9 – 20.7) | 12.0 (7.5 – 18.5) | 0.3673 |
| CCR2 | 65.3 (61.2 – 71.7) | 70.9 (56.1 – 78.7) | 0.4578 | 11.5 (5.6 – 17.6) | 5.8 (2.4 – 12.4) | 0.1369 | 1.5 (1.1 – 2.0) | 1.6 (0.7 – 3.2) | 0.4691 |
| CD80 | 5.1 (1.6 – 14.1) | 1.8 (1.0 – 2.0) | **0.0047** | 0.5 (0.2 – 1.8) | 0.3 (0.2 – 0.5) | **0.0273** | 1.1 (0.4 – 1.5) | 0.5 (0.3 – 1.2) | 0.2806 |
| CD86 | 65.7 (62.8 – 73.4) | 71.2 (58.1 – 79.6) | 0.5005 | 10.4 (5.4 – 15.6) | 6.3 (2.3 – 12.0) | 0.1950 | 14.2 (9.5 – 21.2) | 13.0 (8.9 – 17.2) | 0.3052 |
| TLR3 | 57.9 (52.8 – 63.6) | 60.4 (49.8 – 65.4) | 0.3347 | 10.0 (3.8 – 13.6) | 4.7 (1.8 – 10.6) | **0.0310** | 8.3 (5.1 – 9.7) | 7.1 (3.5 – 11.8) | 0.7788 |
| TLR4 | 0.6 (0.2 – 1.5) | 0.6 (0.2 – 0.8) | 0.4617 | 1.2 (0.5 – 2.3) | 1.1 (0.6 – 1.8) | 0.3395 | 2.0 (0.6 – 2.7) | 1.8 (0.9 – 3.8) | 0.4109 |
| PD-L1 | 0.3 (0.3 – 0.5) | 0.2 (0.1 – 0.2) | **0.0027** | 0.5 (0.2 – 1.2) | 0.5 (0.2 – 1.0) | 0.9873 | 0.5 (0.3 – 1.0) | 0.3 (0.2 – 0.5) | **0.0471** |
| PD-L2 | 0.2 (0.2 – 0.4) | 0.2 (0.1 – 0.2) | **0.0379** | 94.1 (80.0 – 96.5) | 84.7 (49.8 – 94.6) | 0.2342 | 1.1 (0.9 – 2.0) | 1.2 (0.5 – 2.7) | 0.9782 |

Abbreviations: CD=Cluster of differentiation; CCR2= C-C motif chemokine receptor; CL=Classical; IM=Intermediate; MLWH=Mothers living with HIV; MNLWH=Mothers not living with HIV; NC=Non-classical; PD-L=Programmed cell death ligand; TLR=Toll-like receptor

P-Values denoted in bold indicate significance (p<0.05).

Results are presented as median and interquartile range, except when indicated by * in which case results are presented as mean and standard deviation.

**Table S17: Percentage of monocyte marker expression between HIV-exposed and unexposed infants at birth and ten weeks of age**

| **Birth** | | | | | | | | | |
| --- | --- | --- | --- | --- | --- | --- | --- | --- | --- |
|  | **Classical** | | | **Intermediate** | | | **Non-classical** | | |
| **Description** | **HEU (n=29)** | **HUU (n=26)** | **p-Value** | **HEU (n=27)** | **HUU (n=26)** | **p-Value** | **HEU (n=29)** | **HUU (n=26)** | **p-Value** |
| Total % CD14+ CL | 75.6 (64.3 – 88.0) | 82.8 (72.6 – 88.4) | 0.3282 | 11.4 (5.0 – 19.4) | 8.3 (3.4 – 13.3) | 0.5452 | 6.1 (4.6 – 11.5) | 7.9 (5.1 – 9.9) | 0.8794 |
| CCR2 | 74.4 (64.7 – 82.7) | 74.1 (68.2 – 83.5) | 0.8762 | 10.9 (4.6 – 19.3) | 8.1 (3.0 – 12.8) | 0.4767 | 95.2 (89.6 – 100.0) | 96.2 (93.7 – 100.0) | 0.6682 |
| CD80 | 0.8 (0.1 – 1.7) | 0.6 (0.1 – 1.3) | 0.5777 | 0.6 (0.3 – 1.1) | 0.5 (0.2 – 0.8) | 0.4962 | 0.2 (0.1 – 0.3) | 0.3 (0.1 – 0.4) | **0.0467** |
| CD86 | 68.0 (58.1 – 80.4) | 77.1 (66.6 – 85.5) | 0.1617 | 10.9 (4.6 – 19.3) | 8.1 (3.0 – 12.8) | 0.4767 | 5.6 (3.8 – 11.0) | 7.3 (4.1 – 9.8) | 0.8529 |
| TLR3 | 71.3 (63.5 – 78.2) | 73.7 (64.8 – 81.7) | 0.4157 | 9.6 (4.8 – 18.6) | 6.1 (2.3 – 8.9) | **0.0316** | 6.1 (4.6 – 11.5) | 7.9 (5.1 – 9.9) | 0.8794 |
| TLR4 | 4.0 (2.6 – 7.4) | 4.4 (2.4 – 5.9) | 0.9195 | 2.8 (2.1 – 4.5) | 2.4 (1.4 – 2.9) | 0.1608 | 3.0 (1.8 – 5.6) | 3.9 (1.8 – 5.6) | 0.9288 |
| PD-L1 | 2.2 (0.9 – 3.6) | 2.9 (0.8 – 7.9) | 0.3372 | 0.6 (0.2 – 1.8) | 0.4 (0.1 – 1.1) | 0.1742 | 0.0 (0.0 – 0.0) | 0.9 (0.0 – 2.3) | 0.1044 |
| PD-L2 | 3.5 (1.7 – 56.0) | 34.5 (11.7 – 95.5) | 0.2030 | 52.8 (8.4)* | 62.1 (30.4)* | 0.4642 | 64.7 (22.5)* | 77.0 (17.2)* | 0.3253 |
| **Ten Weeks** | | | | | | | | | |
|  | **Classical** | | | **Intermediate** | | | **Non-classical** | | |
| **Description** | **HEU (n=18)** | **HUU (n=20)** | **p-Value** | **HEU (n=18)** | **HUU (n=19)** | **p-Value** | **HEU (n=19)** | **HUU (n=18)** | **p-Value** |
| Total % CD14+ CL | 72.3 (63.5 – 76.4) | 64.3 (56.8 – 74.7) | 0.1213 | 1.9 (1.0 – 2.6) | 2.4 (0.6 – 3.2) | 0.3160 | 25.2 (22.0 – 33.1) | 29.9 (18.4 – 34.5) | 0.5038 |
| CCR2 | 70.5 (62.2 – 72.0) | 59.8 (54.1 – 70.0) | **0.0444** | 0.20 (0.04 – 0.70) | 0.50 (0.03 – 0.90) | 0.3281 | 0.5 (0.3 – 1.1) | 1.2 (0.3 – 1.4) | 0.3113 |
| CD80 | 27.9 (16.7 – 31.3) | 21.0 (17.4 – 25.0) | 0.1223 | 0.8 (0.3 – 1.0) | 0.6 (0.3 – 1.2) | 0.9417 | 6.2 (4.3 – 8.3) | 5.6 (3.9 – 70.1) | 0.8368 |
| CD86 | 63.7 (55.4 – 69.3) | 50.7 (44.1 – 64.7) | **0.0078** | 1.9 (1.0 – 2.8) | 3.0 (1.4 – 3.4) | **0.0416** | 25.4 (22.8 – 33.4) | 30.5 (21.3 – 37.9) | 0.2794 |
| TLR3 | 53.4 (46.3 – 60.9) | 43.5 (37.2 – 50.9) | **0.0093** | 1.9 (1.0 – 2.8) | 3.0 (1.4 – 3.4) | **0.0416** | 12.3 (5.6 – 15.4) | 13.2 (7.9 – 15.0) | 0.6127 |
| TLR4 | 0.7 (0.6 – 1.1) | 1.9 (1.0 – 2.7) | **0.0143** | 0.7 (0.4 – 0.8) | 0.9 (0.4 – 1.5) | 0.1609 | 12.3 (5.6 – 15.4) | 13.2 (7.9 – 15.0) | 0.6127 |
| PD-L1 | 2.5 (1.0 – 3.8) | 2.5 (1.6 – 4.0) | 0.5809 | 0.08 (0.04 – 0.22) | 0.08 (0.06 – 0.17) | 0.7987 | 1.5 (0.4 – 2.6) | 1.0 (0.5 – 2.1) | 0.6127 |
| PD-L2 | 0.7 (0.3 – 1.1) | 0.8 (0.4 – 1.5) | 0.3873 | 0.0 (0.0 – 14.9) | 1.7 (0.0 – 3.9) | 0.7248 | 0.7 (0.2 – 1.2) | 0.9 (0.2 – 1.4) | 0.7187 |

Abbreviations: CD=Cluster of differentiation; CCR2= C-C motif chemokine receptor; CL=Classical; HEU=HIV-exposed-uninfected; HUU=HIV-unexposed-uninfected; IM=Intermediate; NC=Non-classical; PD-L=Programmed cell death ligand; TLR=Toll-like receptor

P-Values denoted in bold indicate significance (p<0.05)

Results are presented as median and interquartile range, except when indicated by * in which case results are presented as mean and standard deviation.

**Table S18: Percentage of monocyte marker expression between HIV-exposed and unexposed infants at six months of age**

| **Six months** | | | | | | | | | |
| --- | --- | --- | --- | --- | --- | --- | --- | --- | --- |
|  | **Classical** | | | **Intermediate** | | | **Non-classical** | | |
| **Description** | **HEU (n=17)** | **HUU (n=16)** | **p-Value** | **HEU (n=15)** | **HUU (n=14)** | **p-Value** | **HEU (n=17)** | **HUU (n=16)** | **p-Value** |
| Total % CD14+ CL | 70.1 (7.6)* | 64.4 (13.8)* | 0.2962 | 1.4 (0.8 – 1.9) | 1.9 (0.9 – 3.8) | 0.2386 | 25.6 (8.4)* | 29.4 (10.8)* | 0.3490 |
| CCR2 | 61.6 (58.0 – 66.0) | 59.0 (49.5 – 68.5) | 0.3633 | 1.4 (0.8 – 1.9) | 1.9 (0.9 – 3.8) | 0.2386 | 1.5 (0.9 – 2.6) | 2.0 (1.6 – 2.6) | 0.4388 |
| CD80 | 9.2 (5.3)* | 7.8 (6.2)* | 0.3347 | 0.5 (0.1 – 0.8) | 0.4 (0.2 – 1.5) | 0.2818 | 6.1 (3.6 – 9.7) | 6.6 (3.4 – 10.9) | 0.8360 |
| CD86 | 55.6 (9.7)* | 57.7 (13.7)* | 0.3697 | 1.4 (0.8 – 1.9) | 1.9 (0.9 – 3.8) | 0.2386 | 26.1 (22.0 – 31.9) | 31.0 (18.9 – 37.7) | 0.3490 |
| TLR3 | 60.1 (56.9 – 64.2) | 57.5 (46.9 – 65.3) | 0.3604 | 1.4 (0.8 – 1.9) | 1.9 (0.9 – 3.8) | 0.2386 | 12.7 (10.0 – 21.9) | 17.8 (12.1 – 23.3) | 0.4074 |
| TLR4 | 0.6 (0.1 – 2.5) | 0.5 (0.3 – 1.3) | 0.7856 | 1.0 (0.3 – 1.3) | 0.9 (0.3 – 2.5) | 0.2990 | 8.5 (7.0 – 21.2) | 12.2 (8.7 – 18.0) | 0.9248 |
| PD-L1 | 0.4 (0.3 – 0.8) | 1.1 (0.4 – 1.7) | 0.1910 | 0.3 (0.2 – 0.4) | 0.4 (0.2 – 1.0) | 0.4998 | 1.1 (0.3 – 2.4) | 1.3 (0.5 – 3.3) | 0.4168 |
| PD-L2 | 0.2 (0.1 – 0.3) | 0.2 (0.1 – 0.7) | 0.2565 | 0.5 (0.1 – 0.8) | 0.4 (0.2 – 1.5) | 0.2818 | 0.6 (0.1 – 1.0) | 0.7 (0.3 – 2.1) | 0.2902 |

Abbreviations: CD=Cluster of differentiation; CCR2= C-C motif chemokine receptor; CL=Classical; HEU=HIV-exposed-uninfected; HUU=HIV-unexposed-uninfected; IM=Intermediate; NC=Non-classical; PD-L=Programmed cell death ligand; TLR=Toll-like receptor

Results are presented as median and interquartile range, except when indicated by * in which case results are presented as mean and standard deviation.

###### **Table S19: Comparison of the cytokine/chemokine data between the two cohorts** **of mothers at 28 weeks’ gestation**

| **Cytokine** | **MLWH: Median (IQR) (pg/mL)** | **MNLWH: Median (IQR) (pg/mL)** | **p-Value** |
| --- | --- | --- | --- |
| GM-CSF | 0.19 (0.19 – 0.19) | 0.19 (0.19 – 0.19) | 0.9328 |
| IFN-γ | 1.48 (0.93 – 2.21) | 1.57 (1.30 – 3.20) | 0.3582 |
| IL-2 | 0.38 (0.18 – 0.69) | 0.56 (0.31 – 0.80) | 0.3626 |
| IL-4 | 0.39 (0.29 – 0.60) | 0.63 (0.39 – 0.86) | 0.1313 |
| IL-6 | 1.36 (0.91 – 1.72) | 1.02 (0.73 – 2.16) | 0.2447 |
| IL-8 | 1.32 (0.98 – 2.45) | 1.37 (0.85 – 1.93) | 0.6190 |
| IL-10 | 3.21 (0.69 – 8.54) | 3.21 (1.24 – 4.77) | 0.9597 |
| TNF-α | 15.7 (10.38 – 21.79) | 13.6 (9.62 – 21.04) | 0.4184 |

Abbreviations: GM-CSF=Granulocyte-macrophage colony-stimulating factor; IFN=Interferon; IL=Interleukin; IQR=Interquartile range; MLWH=Mothers living with HIV; MNLWH=Mothers not living with HIV; TNF=Tumour necrosis factor

###### **Table S20: Comparison of cytokine/chemokine data between the two cohorts of** **infants at ten weeks and six months of age**

|  | **Ten Weeks** | | | **Six Months** | | |
| --- | --- | --- | --- | --- | --- | --- |
| **Cytokine** | **HEU: Median (IQR) (pg/mL)** | **HUU: Median (IQR) (pg/mL)** | **p-Value** | **HEU: Median (IQR) (pg/mL)** | **HUU: Median (IQR) (pg/mL)** | **p-Value** |
| GM-CSF | 0.04 (0.04 – 0.04) | 0.04 (0.04 – 0.04) | 0.9841 | 0.04 (0.04 – 0.41) | 0.04 (0.04 – 0.04) | 0.9841 |
| IFN-γ | 4.95 (2.33 – 7.82) | 5.62 (3.31 – 14.4) | 0.4749 | 3.31 (0.08 – 5.62) | 4.72 (2.33 – 9.52) | 0.4749 |
| IL-2 | 0.01 (0.01 – 0.18) | 0.07 (0.01 – 0.18) | 0.3509 | 0.01 (0.01 – 0.01) | 0.01 (0.01 – 0.01) | 0.3509 |
| IL-4 | 0.77 (0.56 – 0.94) | 0.56 (0.49 – 0.71) | 0.1530 | 0.38 (0.19 – 0.53) | 0.41 (0.19 – 0.56) | 0.1530 |
| IL-6 | 0.61 (0.41 – 1.40) | 0.80 (0.61 – 1.20) | 0.6058 | 1.10 (0.41 – 2.82) | 0.90 (0.65 – 1.60) | 0.6058 |
| IL-8 | 4.30 (2.71 – 6.42) | 2.52 (1.39 – 4.38) | 0.1216 | 4.12 (1.29 – 7.10) | 2.11 (0.64 – 14.68) | 0.1216 |
| IL-10 | 1.81 (1.14 – 3.70) | 3.44 (2.11 – 4.62) | 0.1771 | 1.59 (1.09 – 2.29) | 2.11 (1.23 – 3.52) | 0.1771 |
| TNF-α | 23.07 (15.64 – 26.08) | 24.57 (20.08 – 34.47) | 0.5784 | 24.01 (19.70 – 35.24) | 23.82 (21.19 – 26.46) | 0.5784 |

Abbreviations: GM-CSF=Granulocyte-macrophage colony-stimulating factor; HEU=HIV-exposed-uninfected; HIV=Human immunodeficiency virus HUU=HIV-unexposed-uninfected; IFN=Interferon; IL=Interleukin; IQR=Interquartile range; TNF=Tumour necrosis factor

**Table S21: Comparison of C-reactive protein data between mothers and infants**

| **Mothers at 28 Weeks’ gestation** | | |
| --- | --- | --- |
| **MLWH: Median (IQR) (mg/L)** | **MNLWH: Median (IQR) (mg/L)** | **p-Value** |
| 5.17 (4.24 – 7.30) | 4.16 (1.84 – 6.97) | 0.0510 |
| **Infants at ten weeks of age** | | |
| **HEU: Median (IQR) (mg/L)** | **HUU: Median (IQR) (mg/L)** | **p-Value** |
| 0.15 (0.15 – 2.89) | 1.20 (0.15 – 1.78) | 0.0911 |
| **Infants at six months of age** | | |
| **HEU: Median (IQR) (mg/L)** | **HUU: Median (IQR) (mg/L)** | **p-Value** |
| 3.58 (1.57 – 31.60) | 6.35 (1.75 – 38.00) | 0.2491 |

Abbreviations: HEU=HIV-exposed-uninfected; HIV=Human immunodeficiency virus; HUU=HIV-unexposed-uninfected; IQR=Interquartile range; MLWH=Mothers living with HIV; MNLWH=Mothers not living with HIV
